# An enhanced multi-model ensemble learning architecture for robust network intrusion detection

**DOI:** 10.3389/frai.2026.1895608

**Published:** 2026-08-19

**Authors:** Dwarsala Sireesha, Kakelli Anil Kumar

**Affiliations:** School of Computer Science and Engineering, Vellore Institute of Technology, Vellore, India

**Keywords:** ADASYN oversampling, class imbalance handling, ensemble learning, meta-learning, multi-class attack classification, real-time intrusion detection, stacked generalization

## Abstract

Accurately and in real-time identifying advanced cyber-attacks continues to be a serious challenge for modern Network Intrusion Detection Systems (NIDS), especially in situations of highly imbalanced network traffic load and large-scale network attacks. Signature-based and single-model learning methods are typically ineffecive in capturing the complexity of traffic interactions and are not generalizable to new attack patterns. To overcome these limitations, this study introduces an Enhanced Multi-Model Ensemble Network Intrusion Detection System (EME-NIDS), a deep meta-learning system that combines five different heterogeneous learning paradigms, including Convolutional Neural Networks (CNN), Dense Neural Networks, Transformers, XGBoost, and Random Forests. The probabilistic output of the base learners is then condensed into a 220-dimensional meta-feature space and further processed by a five-layer deep meta-learner with approximately 289 k trainable parameters. A large-scale network flow dataset with 703,168 instances was used to assess the learning stability and attack detection of the minority class in the presence of various attacks, with 43 attacks and one attack class (benign) in the dataset. The experimental evaluation provided a detection accuracy of 95.65% and a macro-ROC-AUC score of 99.76%, outperforming all standalone models. Moreover, the proposed framework, which provides an average inference latency of 8.4 ms is suitable for real-time intrusion detection. The robustness, scalability and practical deployment capability of the proposed EME-NIDS framework were confirmed by statistical significance analysis (McNemar’s test, *p* < 0.001) and ablation studies.

## 1 Introduction

This is in direct contrast to traditional networks, where it is comparatively easy for IT security professionals to segregate and disable affected components of the network. The rising popularity of cloud computing, IoT networks, mobile networks, and fast communication networks has increased the number and complexity of operations required for critical digital resources. As attackers develop versatile intrusion methods, NIDS must keep up with this trend to ensure the correct real-time classification of a wide range of attacks. Traditional security solutions and signature-based IDS are still effective for known threats but often fail when dealing with zero-day attacks, polymorphic malware, and different variants with varying behaviors. Although ML and DL techniques improve the power of NIDS single-model approaches, they lack the ability to generalize and manage high-dimensional traffic patterns, identify rare categories, and struggle with significant imbalances in multi-class security datasets. This necessitates the development of more diverse and ensemble-based intrusion detection systems for these networks. Recent studies have increasingly focused on multi-ensemble-driven NIDS that employ complementary learning frameworks to enhance precision and resilience. For example, Gupta et al. (2025) suggested an ML-DL approach for multi-class attack detection in the NSL-KDD and CICIDS-2017 datasets, and excellent results were obtained by applying an ensemble of RNN, LSTM, and XGBoost. Saravanan and Prasad (2025) created an Ensemble Deep Multi-Model Algorithm incorporating CNN, LSTM and Transformer layers to examine user behavior within networks.

In addition, NIDS ensemble learning has been utilized for niche applications. Pandeeswari and Preethi (2025) proposed three models, VCNN, VCANN, and PAAE, which achieved 99.22% accuracy in botnet usage identification. These models were, specialized for botnet-based use cases, not for broader multi-class traffic. Raza and Muzammil (2023) investigated ML-DL ensembles using the HIKARI-2021 dataset and achieved an accuracy of 96.32. However, their research primarily focused on anomaly-based detection. It does not extensively cover multi-class classifications. Learning-based Methods Learning mechanisms have also been scrutinized for multi-attack classification, as reported by Silivery et al. (2023), who tested LSTM-RNN, RNN, and DNN models on the KDD’99 NSL-KDD and UNSW-NB15 datasets. Their approach is suitable for temporal dependencies, which do not account for fusion at the ensemble level, and exhibit poor scalability. Parvathy et al. (2023) conducted a survey on application-layer protocol attacks and ransomware threats in IoT, and provided a taxonomy of attack vectors across various layers of IoT. Their analysis supports the conclusion that application-layer exploits are common and feature sets that encode protocol and application semantics are needed. While no single detection model is shown here, in this survey the reader will find some interesting options for low-level flow and higher order semantics detection, and some insight into why EME-NIDS incorporates protocol-aware features as well as various model types.

These studies demonstrate the promise of ensemble learning for NIDS; however, numerous research gaps remain. Numerous earlier investigations are constrained by datasets that show limited generalizability across different attack types and lack sophisticated approaches to address class imbalance issues. Moreover, only a few studies have utilized meta-learning to optimize model fusion. Additionally, few studies have achieved real-time inference speeds for practical deployment in high-throughput enterprise networks. To overcome these limitations, this study proposes a scalable, high-performance intrusion detection framework that integrates multiple learning paradigms into a deep meta-learning architecture with ensemble methods to enable superior detection accuracy across 44 attack classes in a large-scale, highly imbalanced dataset used in previous studies.

### 1.1 Research contributions

The objective of this study is to present the EME-NIDS: Enhanced Multi-Model Ensemble Network Intrusion Detection System that can address such limitations mentioned in literature. The main contributions of this study are as follows:

- *Novelty meta-learning architecture*: Our proposed EME-NIDS framework has novelty in its architecture as compared to the existing frameworks in terms of: (1) Scale of Classification: The existing stacking based ensemble models such as EDMMA (Saravanan and Prasad, 2025), and Random Forest (Pear and Kibria, 2024) have implemented using maximum of 25 benign and attack classes where as our proposed EME-NIDS is implemented using 703,168 flow samples with 44 benign and attack classes. (2) Meta-Learner: The meta-learner of proposed EME-NIDS used 289,000 trainable parameters, instead of fixed-weight voting (Ullah et al., 2025), or static rank-based combination using CFA (Owusu et al., 2025). Our meta-learner is trained efficiently on each attack class with learning optimal fusion of weights for each class. (3) Model Diversity: Heterogeneous deep-learning models such as CNN, Dense Network, Transformer and machine leaning models such as XGBoost, and Random Forest have integrated without errors in the proposed EME-NIDS and achieved high attack detection accuracy as compared to the existing ensemble frameworks.
- *Advanced preprocessing pipeline*: To combat the severe class imbalance, we performed ADASYN-based class balancing, Standard Scaler normalization, outlier elimination, and statistical feature selection techniques in an attempt to alleviate it.
- *Extensive experimental validation*: A robust evaluation using 5-fold stratified cross-validation, ablation studies, and McNemar’s statistical significance testing showed significant improvements over the use of individual models and other state-of-the-art ensemble methods.
- *Real-world applicability*: Our proposed architecture achieved low inference time and suitable for deployment in real world applications such as IOT threat detection, Enterprise IDS and Cloud Security Monitoring etc.

The rest of this paper is organized as follows: Section 2 reviews the existing literature on machine learning, deep-learning and ensemble-based Network Detection Systems which are the gaps addressed in this proposed research. The proposed methodology consists of data preprocessing procedures, design of the single base models and deep meta-learning ensemble architecture, depending on the type of datasets, measures, and hyperparameter settings, which are presented in Section 3. The results of various experiments are summarized in Section 4. These include performance analysis, ablation studies, and validation and interpretation of interpretability. Section 5 concludes the findings of this study and elaborates on how they can be used for network intrusion detection in the future. Section 6 summarizes and discusses the boundaries of the proposed approach and further research.

## 2 Related work

The studies on intrusion detection have expanded to encompass classic machine learning, deep-learning, and ensemble-based techniques. Prior work can be broadly grouped into three thematic subsections, which are described in detail below, to provide context for the contribution of the EME-NIDS.

### 2.1 Traditional machine learning approaches

The theory of support vector networks (SVN) was proposed by Farhat (1992), where a kernel-based nonlinear mapping was used and the maximum margin solution was obtained, with strong theoretical guarantees for generalization even in very large dimensional feature spaces. The applications of soft-margin optimization and structural risk minimization thus enabled the SVM to successfully make robust classifications on nonlinearly separable and noisy datasets, which formed the basis for SVM being one of the foundational methods in statistical learning theory. Random Forests is one of the most popular ensemble learning methods proposed by Jin et al. (2020) that is based on the random selection of features and bagging method to build uncorrelated decision trees. This procedure greatly improved the accuracy and stability of the model. The ability of Random Forests to be highly resistant to overfitting, to scale efficiently and to provide good estimates of variable importance makes them a significant competitor to kernel-based classifiers for large-scale and high-dimensional data. Kocher and Kumar (2021) provided a comprehensive survey of the use of machine learning and deep-learning techniques for intrusion detection. They observed critical issues such as overfitting, scalability, and the inability of traditional ML models to generalize well for different traffic patterns. Their review clearly indicates that hybrid models are the future of intrusion detection systems. Hence, their work directly justifies the need for combined ML-DL models, such as EME-NIDS. The lack of deep feature representation restricts the performance of complex multi-class intrusion datasets. This motivates the integration of deep architectures into ensemble systems. Aljrees (2024) demonstrated that preprocessing, especially using KNN imputation and feature normalization, positively impacts downstream ensemble performance. In this respect, the study used stacking to combine various ML classifiers. Outside the cybersecurity domain, this study validates the importance of preprocessing pipelines, such as the removal of outliers and scaling of features, which EME-NIDS adopts and enhances. Owusu et al. (2025) proposed a cognitive diversity fusion technique called Combinatorial Fusion Analysis (CFA), which aggregates the rank scores of multiple base models to improve the classification of low-profile attacks. Although CFA is effective, it is a static fusion mechanism and lacks the adaptability of meta-learning and deep feature representations. Their findings justify the need for learnable fusion strategies in ensemble systems. Salehpour et al. (2025) information filtering followed by ensemble ranking—followed by a lightweight Random Forest classifier. They are very good in memory-limited applications but lack in the model diversity and generalization. This highlights the importance of scalable ensemble frameworks for effectively dealing with large-dimensional and multi-class intrusion data sets. Chen and Guestrin (2016) introduced a scalable and regularized gradient tree boosting framework, XGBoost, which incrementally combines sparsity-aware split-finding, weighted quantile sketching, and cache-aware optimization for large-scale learning.

### 2.2 Deep learning for network intrusion detection

Zheng et al. (2025) proposed a hybrid intrusion detection system that combined LSTM with autoencoders for LFC systems. This technique achieves more than 99% accuracy with reduced latency; however, its design is inherently coupled with industrial CPS environments and does not scale well in general NIDS scenarios. This study demonstrated the strengths of temporal deep-learning models while highlighting the need for broader applicability. Pear and Kibria (2024) proposed a CNN–LSTM hybrid that learns spatial and temporal patterns in network traffic. Although it performed well, it was at a disadvantage when the classes were highly imbalanced and failed to generalize across different attack distributions. This highlights the importance of deep models combined with other learners to achieve a robust performance (Alblehai, 2025). CNN-GRU hybrid with FW-SMOTE targeted the IoT dataset imbalance. The results approach 99% accuracy, indicating that IoT intrusion detection can be improved using spatial and temporal modeling. However, oversampling may result in synthetic bias, reducing generalization over large and multiclass datasets. This motivates the use of balanced approaches, such as ADASYN, in EME-NIDS. Alkadi et al. (2021) combined blockchain smart contracts with BiLSTM-based intrusion detection to enable secure and tamper-evident collaborative detection. Although this increases its trustworthiness, it comes at a very high computational and latency cost, highlighting the trade-off between the deep-sequence capabilities and scalable efficiency of the model. Hu et al. (2023) proposed an LSTM combined with a self-attention mechanism, trained on the enriched version of the UNSW-NB15 dataset, DNTAD. The attention mechanism helps to re-weight the key temporal segments and improves the accuracy of anomaly detection tasks. However, this model is still a single deep architecture system without sufficient robustness for multi-class tasks. Alshehri et al. (2024) proposed an attention CNN that strengthens IIoT security by applying mutual information based feature filtering. Although the method attained high accuracy and indicated that attention could sharpen feature discrimination, its IIoT-centric focus has limited applicability in broad network applications. Sasi et al. (2024) proposed a SHAP-explained CNN-LSTM-Attention framework with dataset augmentation. This model provides interpretable predictions and improved performance in detecting IoT-attack datasets. However, this model is complex, and its performance suffers in large-scale multiclass IDS. Vishnu et al. (2022) applied self-attention architectures to classify vulnerability descriptions using CTI datasets. They observed that the performance of the attention-based methods outperformed that of the SVM and CNN-LSTM in semantic extraction. Although not a traffic-based IDS, this study corroborates the effectiveness of attention, which justifies the inclusion of transformer-style models in EME-NIDS. Qaddos et al. (2024) proposed a hybrid CNN–GRU architecture for IoT intrusion detection and fortified it with a feature-weighted SMOTE (FW-SMOTE) resampling strategy. On IoTID20 and UNSW-NB15, the model provides very high reported accuracies of ≈99%, proving that such a combination of convolutional spatial filters with recurrent temporal modeling is suitable for IoT-style traffic. Nevertheless, the design is optimized for IoT datasets and relies heavily on oversampling, which can introduce synthetic bias and lower generalization to broad multiclass network datasets. The findings of this study encourages the combination of complementary deep architecture and careful balancing in EME-NIDS and point to the need for a generalizable fusion strategy not just for IoT specific applications. Kumar and Aravind (2024) designed a hybrid cloud intrusion detection system (IDS) model that used 1D-CNN and Informer model optimized by Hunger Game Search Optimization Algorithm. CNN is adept at extracting local traffic features, while the Informer efficiently captures the long-term temporal dependency in traffic data with lower computational complexity. The CNN has the ability to extract the local traffic features while the Informer is efficient in capturing the long-term temporal dependency in the traffic data with lower computation cost. The model has achieved an accuracy of 99.16% on CSE-CIC-IDS2018 dataset, outperforming conventional deep-learning methods. The results of this work show that a combination of convolutional, transformer-based and metaheuristic optimization methods can be applied to a scalable cloud IDS.

### 2.3 Ensemble learning for intrusion detection

Farhat et al. (2025) introduced a dynamic classifier selection mechanism that selects the best model for each attack type. They attained an accuracy of > 99% using SMOTE and XAI integration. However, their work performed selection and not ensemble fusion, hence losing the synergistic benefits of combining multiple models. Ahmad et al. (2025) combined Random Autoencoders, Isolation Forest, and Random Forest via a meta-classifier while integrating Differential Privacy and Deep Q-Learning for adaptive policy control. Their method is powerful for anomaly detection but computationally heavy; hence, it is not suitable for large multi-class NIDS. Misra and Mishra (2025) combined ML models (XGBoost, RF, and LightGBM) with BERT embeddings of malware for threat scoring. Their hybrid architecture showed a significant improvement in detection accuracy. However, it was designed specifically for malware classification, rather than network intrusion detection. Al-Quayed et al. (2024) develop an ensemble using Decision Trees, MLPs, and Autoencoders on WSN intrusion detection, achieving near 99.5% accuracy. Although the system is effective, it is highly domain-specific and lacks advanced ensemble fusion mechanisms, such as meta-learning. Aljuhani et al. (2024) design an IoMT-oriented IDS by using PSO-based feature selection in combination with ML/DL ensemble models and SHAP explainability. The model shows good performance in highly constrained environments but cannot be scaled up to diverse types of attacks. Zeeshan et al. (2022) performed flow and protocol metadata fusion using deep neural networks to detect DoS/DDoS attacks. Although effective, this work is restricted to a narrow attack spectrum and does not generalize to large multi-class IDS tasks. Ullah et al. (2025) concatenate CNN, BiLSTM, Random Forest and Logistic Regression input through soft voting. Although high-performance results are obtained over different IoT datasets, the fixed weights, as opposed to learnable meta-fusion, hinder adaptability. This survey provides a taxonomy of IoT application-layer threats, emphasizing the importance of protocol-aware features and a multi-layered IDS design. Although it did not provide any model, it highlighted the need for diverse feature sets, thus validating the multi-feature design of the EME-NIDS.

### 2.4 State-of-the-art comparison

Table 1 compares the proposed model with recent state-of-the-art Network Intrusion Detection System (NIDS) approaches published in the last 3 years (2024–2026). The comparison shows different datasets, the number of classes, and the classification accuracy published in the literature for each class. Although the accuracies of these existing approaches vary from 90.17 to 97.50% on different benchmark datasets, it is difficult to compare them directly because of the differences in datasets and class distribution. The proposed approach achieves 95.6% accuracy on the Enhanced Dataset of 44 attack classes and is competitive with current NIDS models. Overall, the results show that the proposed framework can achieve a good balance of detection accuracy and multi-class intrusion classification capability.

**Table 1:** Performance comparison of state-of-the-art NIDS approaches.

Method	Year of published	Dataset	Classes	Accuracy
BOT-MMEL-SSNI (Pandeeswari and Preethi, 2025)	2025	BoT-IoT	Various	94.56%
Random Forest (with resampling) (Pear and Kibria, 2024)	2024	UNSW-NB15	6	90.17
EDMMA (Saravanan and Prasad, 2025)	2025	UNSW-NB15	25	97.50%
Enhanced Model (Harahsheh et al., 2024)	2024	BoT-IoT	Various	92.0%
		N-BaIoT	115	93.0%
		ToN_IoT	47	94.0%
		CICIDS 2017	80	93.0%
		NSL-KDD	41	92.0%
Random Forest (Wali et al., 2024)	2024	UMNIDS	-	92%
EME-NIDS	2026	**Enhanced dataset (UMNIDS)**	**44**	**95.6%**

Bold values indicate better performance of the proposed EME-NIDS framework in comparison to the state-of-the-art methods.

## 3 Methodology

**Figure 1:**
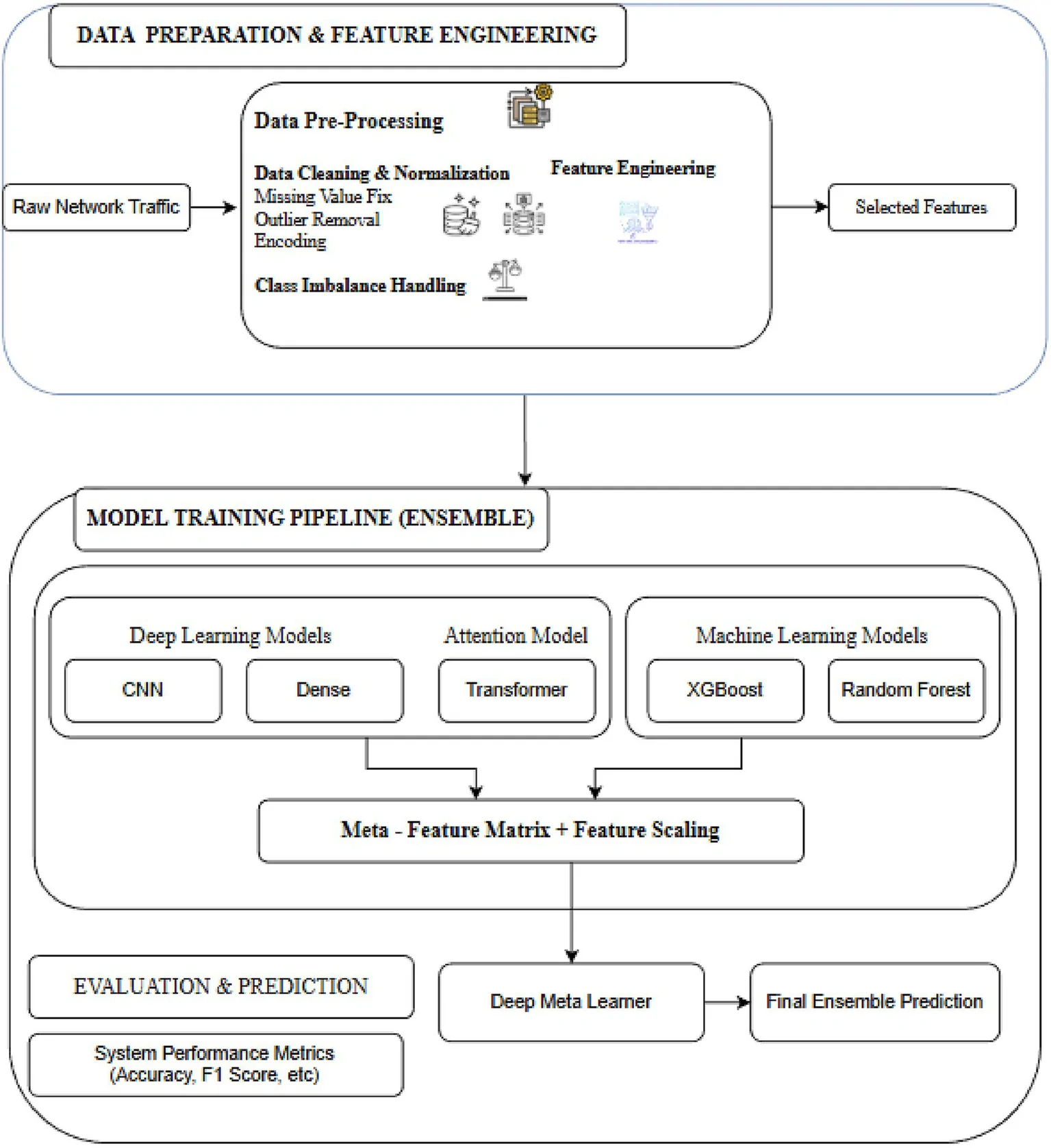
Architecture of the proposed EME-NIDS model.

In the Enhanced Multi-Model Ensemble Network Intrusion Detection System (EME-NIDS), the core motivation is the high degree of precision and real-time detection of 44 categories of various attacks by combining different learning methods within a single ensemble. Therefore, this method can be divided into four major logical steps: characterization and preprocessing of data; construction of a deep-learning and machine learning base model mixture; construction of a deep meta-learning ensemble; and formation of a strong training regime with hyperparameter tuning and detailed validation. Each component in itself is designed in such a way that it encourages other elements to be scalable, generalized, and robust against class imbalance, a common signature of modern network intrusion datasets. The subsequent sections discuss each methodological component in detail. Figure 1 depicts the end-to-end architecture of the proposed Enhanced Multi-Model Ensemble Network Intrusion Detection System. It integrates data preprocessing, feature engineering, multi-model training, meta-feature fusion, and deep meta-learning into a single framework. The architecture demonstrates how deep-learning and traditional machine learning models collaboratively enhance the detection accuracy across complex and imbalanced network traffic scenarios.

### 3.1 Dataset description and characteristics

In this study, we used the Unified Multimodal Network Intrusion Detection Systems dataset (UM-NIDS) which is publicly available from IEEE DataPort (DOI: 10.21227/d8at-gb29; Wali et al., 2024). This dataset combines network traffic from benchmark datasets (CICIDS-2017, CICIoT-2023, UNSW-NB15, and CIC-DDOS2019) to create a multimodal dataset comprising 703,168 network flow samples labeled with 44 classes of traffic (43 classes of attacks and 1 class of BENIGN). The dataset is licensed under Creative Commons Attribution 4.0 International License (CC BY 4.0) and is freely available for research. Throughout this work, we refer to it as the ‘Enhanced NIDS Dataset.

Table 2 shows that, sources of data for UM-NIDS used in this study (Wali et al., 2024). The combined data set contains 703,168 samples, including a stratified subset that are combined from these source data sets. The licenses listed are the terms governing the availability of all source datasets.

**Table 2:** UM-NIDS dataset with Sub-Datasets.

Source dataset	Primary attack types	Approx. samples
CICIDS-2017	DoS, DDoS, BruteForce, infiltration, botnet	~2.8 M
CICIoT-2023	IoT-specific DDoS/DoS, Mirai variants	~4.6 M
UNSW-NB15	Exploits, backdoor, reconnaissance, generic	~2.5 M
CIC-DDoS 2019	Botnet, DoS, DDoS.	~73 M
UM-NIDS (merged)	All 43 attack types + BENIGN	703,168 (sampled)

**Figure 2:**
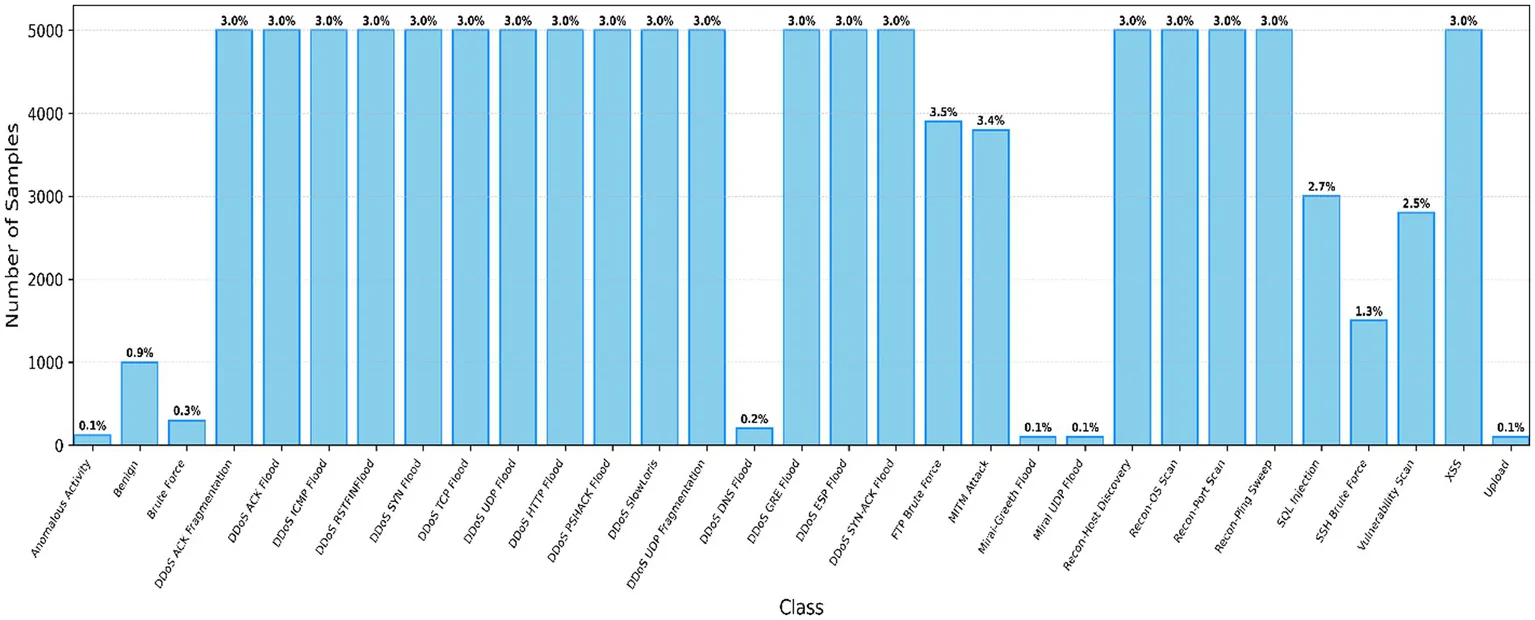
Class distribution of the UM-NIDS dataset before ADASYN oversampling.

Figure 2 presents the class distribution with multi-class imbalance of 43 attack classes and one benign class. Few DDoS classes had around 5,000 instances (around 3%), and hardly any classes had SQL Injection, Worms, Shellcode, Heartbleed, and Infiltration with less than 0.1% instances. This explains why the ADASYN and class weight approaches must be used during the preprocessing phase. The bar chart also reveals that benign traffic is only 3.0%, and the major attack families are DDoS, Reconnaissance and Exploits, thus highlighting the importance of a proper classification system to process both major and minor classes. This is one of the main challenges in intrusion detection and directly motivates the ensemble-based architecture of EME-NIDS. Table 3, the enhanced NIDS dataset is composed of 703,168 labeled flow samples with 78 engineered features, 43 attack categories and one benign traffic class (44 classes total). These features capture a wide range of flow-level behavioral attributes, including packet statistics, flow durations, inter-arrival times, and traffic rates, among protocol-level attributes. Of the 78 features, 75 were numerical and 3 were categorical, making the dataset suitable for both deep-learning and traditional machine learning pipelines. Another strong characteristic of this dataset is the huge class imbalance, as the rarest attack class has a 1:847 ratio relative to the majority of benign traffic. This causes substantial problems when training the model for a high recall of minority attack types. To ensure that the model was evaluated reliably and without any bias, the dataset was split using a stratified 60–20–20% train–validation–test split such that class-wise proportionality was maintained across all splits. The realistic distribution of the real-world network behavior of the dataset is shown in Table 4, with BENIGN traffic occupying 77.9%, followed by DDoS, which accounts for 18.2%, PortScan, which accounts for 2.3%, Bot 1.0%, and a set of rare attacks such as SQL Injection, Heartbleed, and Infiltration accounting for less than 0.1%. These characteristics drive the design of the proposed preprocessing pipeline, model architecture, and ensemble learning strategy.

**Table 3:** Dataset statistics and class distribution.

Metric	Value
Total samples	703,168
Features	78
Classes	44
Training samples	421,901 (60%)
Validation samples	140,634 (20%)
Test samples	140,633 (20%)
Class balance ratio	1:847 (most imbalanced)
Missing values	0% (after preprocessing)
Categorical features	3
Numerical features	75

**Table 4:** Top 10 classes in enhanced UM-NIDS dataset.

Attack class	Frequency	Percentage	Category
BENIGN	547,832	77.9%	Normal
DDoS	128,027	18.2%	DoS
PortScan	15,892	2.3%	Reconnaissance
Bot	7,255	1.0%	Malware
DoS slowloris	5,796	0.8%	DoS
Web Attack	2,180	0.3%	Web-based
Brute Force	1,507	0.2%	Password Attack
SQL Injection	21	<0.1%	Injection
Infiltration	36	<0.1%	Penetration
Heartbleed	11	<0.1%	Vulnerability

### 3.2 Data preprocessing pipeline

A systematic six-stage preprocessing pipeline was developed to ensure strong model training, good performance evaluation, and prevention of information leakage. The large volume of data and the highly imbalanced and heterogeneous nature of network intrusion events mean that there were a number of pre-processing steps that were carefully designed to ensure the integrity of the experimental evaluation process, while maintaining the quality of the data. First, the data set was split via stratified sampling into training, validation and test sets with 60, 20, and 20%, respectively. Stratification maintained the original class distribution for all partitions, thus maintaining representative samples of both majority and minority attack categories throughout the learning process. Second, the values that were missing in the training data were filled in using a method called median imputation, which fills in missing values in a way that still maintains the statistical distribution of the features, but with less influence from extreme values. These imputation parameters were then used to impute the validation and testing sets. Third, outlier detection and elimination was carried out using Interquartile Range (IQR). To minimize noise and enhance model stability, samples that were more than 1.5 × IQR from the first and third quartiles were considered outliers and excluded. Fourth, for all numerical attributes, the StandardScaler method was used to transform the attributes into zero-mean and unit-variance. To avoid leakage information, the scaling parameters used were fitted only to the training set and then applied to the validation and testing sets with the learned scaling. For models such as CNNs, Dense Networks, and Transformers, which rely on gradients for optimization, standardization was especially crucial, helping to stabilize optimization and speed convergence.

Fifth, categorical attributes were transformed using Label Encoding followed by ANOVA statistical feature selection. These methods were used to remove redundant features and low throughput to reduce overfitting and model complexity. Only the training data were used to fit the feature selection method, and the resulting selection was applied to the validation and testing subsets. Sixth, the class imbalance issue was handled by implementing Adaptive Synthetic Sampling (ADASYN) algorithm. ADASYN produces synthetic minority instances according to the local density of the data, thus increasingly focusing on the hard-to-learn areas of the feature space. Only the training subset after data partitioning and feature preprocessing was oversampled to obtain unbiased evaluation. No samples were used for validation or testing in the synthesis of samples. In addition, data processing, such as scaling and feature selection, as well as ADASYN oversampling, were applied separately to each training fold in the 5-fold stratified cross validated process. The validation fold was not used at all in the preprocessing or training of the model, thus without any data leakage. The resulting preprocessing pipeline yielded a noise reduced, balanced, and standardized data set that could be used for machine learning and deep-learning techniques. Then the probabilistic outputs of the CNN, Dense Network, Transformer, XGBoost, and Random Forest base learners were combined to form a 220-dimensional meta-feature space as input to the proposed deep meta-learning ensemble architecture.

#### 3.2.1 Feature engineering procedure

The proposed Enhanced Meta-Ensemble Network Intrusion Detection System (EME-NIDS) does not add any handcrafted or automatically generated features. Rather, it simply utilizes the 78 flow-level features that are included within the original UM-NIDS dataset. They cover all types of network traffic information, ranging from statistical flow-based measurements to various packet-based attributes and protocol information, temporal patterns, and statistics of traffic rates. No additional feature extraction or generation was necessary in this study as these features were already carefully designed and validated in the construction of the UM-NIDS. A well-designed feature engineering pipeline was developed before training models to ensure reliable model training and improve data quality. The parameter estimation preprocessing steps (scaling, features selection, oversampling) were applied in each fold of the 5-fold stratified cross-validation method but only to the training fold. This guarantees that all information in the validation and test sets were not used to affect the pre-processing.

**Figure 3:**
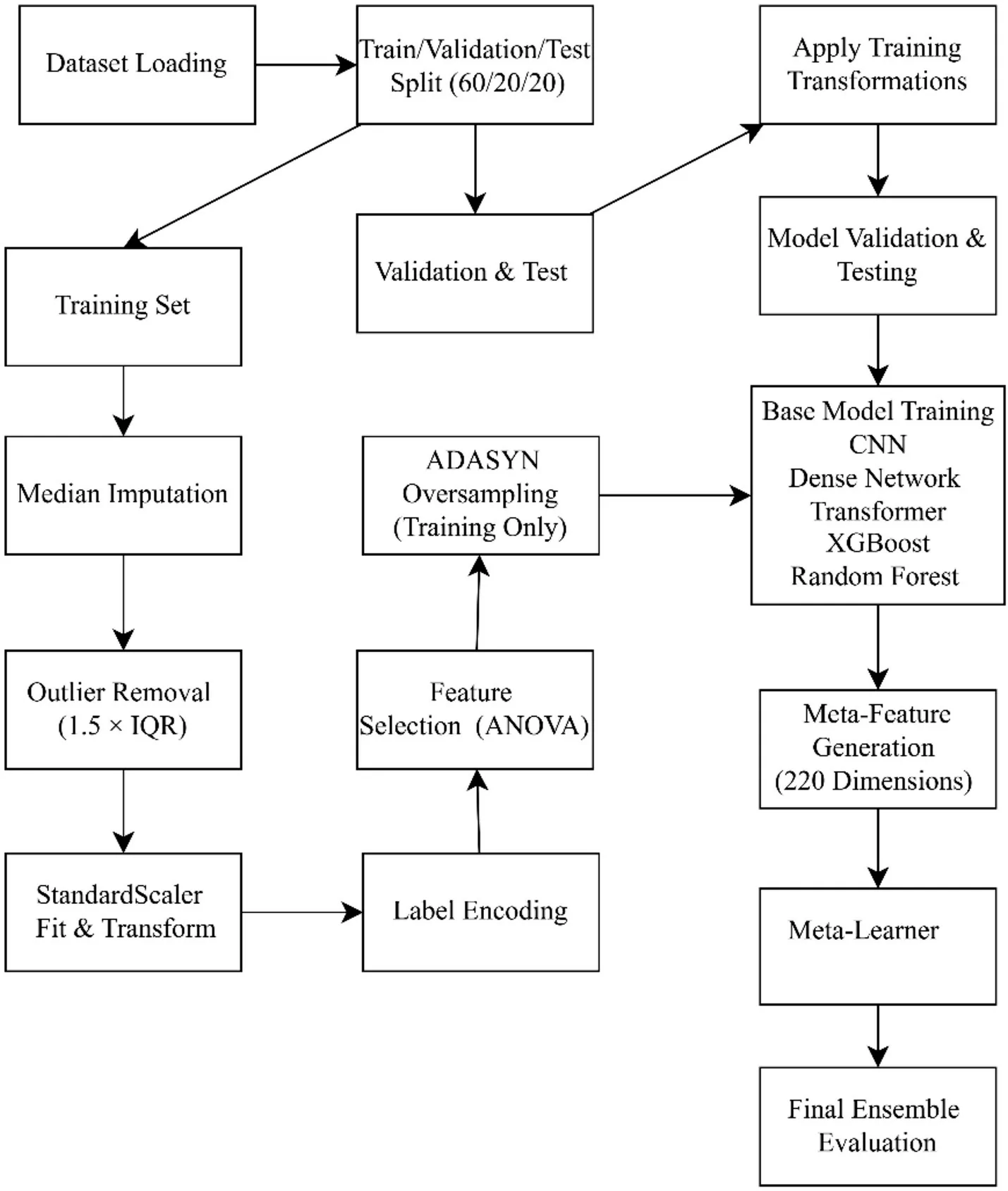
Six stages of the data preprocessing pipeline of the EME-NIDS framework.

Table 5 shows the feature engineering process used for this work. In each stage, the quality of the features are enhanced with minimization of redundancy among features. The standardized features are input to the learning models with sequence of operations to reduce the bias during model training phase. Figure 3 illustrates the experimental workflow of the proposed EME-NIDS with comprehensive leakage free process. The data is stratified sampled to partition into training, validation and test sets. All data pre-processing is done only on the training data to avoid information leakage, such as median imputation, outlier removal, feature scaling, label encoding, feature selection, and ADASYN oversampling. The processed data is then fed to five different base learners (CNN, Dense Network, Transformer, XGBoost, and Random Forest) whose individual output is combined into a 220-dimensional meta-feature space, for deep Meta-learning and final ensemble evaluation. First, the dataset was divided into training, validation, and testing sets to avoid information leakage. All the preprocessing steps, such as median imputation, outlier removal, feature scaling, feature selection, and ADASYN oversampling were fitted only to the training data. The learned transformations were then applied to the validation set and testing set without recalibration. The preprocessing and ADASYN were performed independently in each training fold during 5-fold cross validation for the validation folds.

**Table 5:** Summary of feature engineering.

Step	Technique	Purpose	Effect on feature space
1	Stratified Data Partitioning	Preserve class distribution	Representative training, validation, and testing subsets
2	Median Imputation	Handle missing values	Complete feature vectors without distribution distortion
3	IQR Outlier Removal	Eliminate noisy observations	Improved robustness and reduced variance
4	StandardScaler	Standardize numerical features	Zero mean and unit variance for stable optimization
5	Label Encoding	Convert categorical features	Numerical representation for statistical learning
6	ANOVA Feature Selection	Remove redundant and low-information attributes	Reduced dimensionality and improved discriminative capability
7	ADASYN Oversampling	Balance minority attack classes	Improved minority-class representation without data leakage

### 3.3 Proposed EME-NIDS Model

The EME-NIDS model built using five heterogeneous models namely CNN, Dense Network, Transformer, XGBoost, and Random Forest to characterize complex and heterogeneous network traffic. Each model has unique learning capabilities to enhance ensemble diversity as shown in Table 6. CNNs models can effectively learn the localized hierarchical traffic patterns and spatial correlations of neighboring network features. Dense Neural Network is used to find global nonlinear relationships between network attributes without the assumption of spatial locality. Multi-head self-attention of the transformer model allows the processing long-range dependencies and contextual interactions which are not considered by convolutional models. These deep-learning models are complemented by the inclusion of tree-based machine learning models such as XGBoost and Random Forest to achieve better performance over intrusion detection datasets. XGBoost builds decision trees using gradient boosting to reduce the prediction errors with high discriminative decision boundary. Random Forest constructs a series of decision trees using bootstrap aggregation and random selection of features, which further reduces the variance of the prediction and provides robustness against overfitting. Consequently, these two models exhibited complementary prediction behaviors. The proposed ensemble can simultaneously learn the spatial, nonlinear, contextual, and tree representations of intrusion network traffic simultaneously. The probabilistic outputs of these different learners are combined using deep meta-learning architecture with adaptive combination strategies instead of using static voting or static model weights. Our mechanism results robust EME-NIDS ensemble model which enables efficient detection of minority attack classes and minimizes correlated prediction errors.

**Table 6:** Learning capabilities of EME-NIDS ensemble model.

Model	Learning capability	Contribution
CNN	Local spatial feature extraction	Captures hierarchical traffic signatures
Dense Neural Network	Global nonlinear representation	Complements CNN by modelling global feature interactions
Transformer	Attention-based contextual learning	Captures complex feature interactions that CNN may overlook
XGBoost	Gradient boosting	Produces discriminative decision boundaries and reliable probability estimates
Random Forest	Bootstrap aggregation	Improves robustness and complements XGBoost through diversity

#### 3.3.1 Convolutional neural network

The Convolutional Neural Network (CNN) was selected because of its ability to automatically learn localized and hierarchical feature representations from network traffic data. Convolutional filters effectively capture local dependencies among neighboring traffic features, enabling the identification of characteristic intrusion patterns. CNNs are also robust to noise and capable of extracting discriminative nonlinear representations without manual feature engineering and detailed architecture layers, as shown in Table 7.

**Table 7:** CNN architecture of the proposed EME-NIDS framework.

Layer type	Output shape	Parameters	Activation	Regularization
Input	(batch, 78, 1)	0	–	–
Conv1D-1	(batch, 78, 128)	1,024	ReLU	BatchNorm
Conv1D-2	(batch, 78, 128)	114,816	ReLU	BatchNorm
MaxPool1D	(batch, 39, 128)	0	–	Dropout (0.15)
Conv1D-3	(batch, 39, 256)	164,096	ReLU	BatchNorm
Conv1D-4	(batch, 39, 256)	327,936	ReLU	BatchNorm
MaxPool1D	(batch, 19, 256)	0	–	Dropout (0.2)
Conv1D-5	(batch, 19, 512)	393,728	ReLU	Batch Norm
Conv1D-6	(batch, 19, 512)	786,944	ReLU	BatchNorm
GlobalAvgPool	(batch, 512)	0	–	Dropout (0.25)
Dense-1	(batch, 512)	262,656	ReLU	BatchNorm + Dropout (0.4)
Dense-2	(batch, 256)	131,328	ReLU	BatchNorm + Dropout (0.3)
Dense-3	(batch, 128)	32,896	ReLU	Dropout (0.2)
Output	(batch, 44)	5,676	Softmax	–
Total		2,221,100		

#### 3.3.2 Dense neural network

The enhanced dense neural network acts as a global feature interaction model, wherein every neuron learns the relationship between all input features. This architecture works particularly well when the features are well-engineered and independent spatial locality. The deep fully connected network architecture for high-dimensional feature learning and nonlinear interaction modeling is presented in Table 8. This demonstrates how the progressive reduction of layers with Batch Normalization and Dropout prevents overfitting and stabilizes the training processes. This allows architecture to generalize well with approximately 1.04 M parameters, making it suitable for large-scale intrusion detection tasks.

**Table 8:** Dense network architecture of the proposed EME-NIDS framework.

Layer	Neurons	Parameters	Activation	Regularization
Input	78	0	–	–
Dense-1	1,024	80,896	ReLU	BatchNorm + Dropout (0.4)
Dense-2	512	524,800	ReLU	BatchNorm + Dropout (0.3)
Dense-3	512	262,656	ReLU	BatchNorm + Dropout (0.3)
Dense-4	256	131,328	ReLU	BatchNorm + Dropout (0.25)
Dense-5	128	32,896	ReLU	BatchNorm + Dropout (0.2)
Dense-6	64	8,256	ReLU	Dropout (0.1)
Output	44	2,860	Softmax	–
Total		1,043,692		

#### 3.3.3 Enhanced transformer network

Finally, the Transformer architecture is used to capture long-range dependencies and global feature interactions through self-attention mechanisms through that the attack pattern can be distributed across multiple, unrelated features which are crucial to intrusion detection.

Table 9 shows the structure of the designed transformer which was used to model long-range dependencies and complex temporal relationships in the network traffic. Combined with an 8-head multi-head self-attention, it can realize parallel contextual learning, and the stacked transformer blocks improve feature abstraction. The architecture has a balanced parameter size of ~2.2 M, which provides a high representational power with reasonable computational resources.

**Table 9:** Transformer architecture of the proposed EME-NIDS framework.

Component	Configuration	Parameters
Embedding	256 dimensions	20,224
Multi-Head Attention	8 heads, 32 key_dim	98,560
Feed Forward	512 → 256 hidden	394,752
Layer Normalization	–	1,536
Transformer Blocks	3 layers	1,486,848
Classification Head	512 → 256 → 128 → 44	198,892
Total parameters		2,200,812

#### 3.3.4 Traditional machine learning models

Traditional ML models are included to provide an interpretable and, efficient baseline and to compare deep-learning performance with classical approaches. As shown in Table 10, the classic machine learning part contains XGBoost and Random Forest. Optimized by a Bayesian hyperparameter search, XGBoost uses 200 estimators of depth-restricted tree structures to yield highly discriminative, decision boundaries. Random Forest adds stability through bootstrap aggregation, which reduces variance and increases generalization.

**Table 10:** Hyperparameters and Optimization Techniques of Machine Learning Models.

Model	Parameter	Value	Optimization method
XGBoost	n_estimators	200	Grid search
	max_depth	8	Bayesian opt
	learning rate	0.1	Random search
	Subsample	0.8	Cross-validation
	colsample_bytree	0.8	Grid search
Random forest	n_estimators	150	Grid search
	max_depth	12	Bayesian opt
	min_samples_split	5	Cross-validation
	min_samples_leaf	2	Grid search
	max_features	‘sqrt’	Domain knowledge

Each base model was trained using 5-fold stratified cross-validation, early stopping, and learning rate scheduling to ensure robustness. Together, they can form a diverse and powerful set of base models for downstream ensemble integration.

### 3.4 Meta-feature learner

After training all five base models, a complete meta-feature matrix was created to support second-order learning (Table 11). Each base model produces a 44-dimensional probability vector, where 43 attack categories and a benign traffic class exist in the classification framework. The proposed classification framework consists of 44 output classes (including one benign traffic class and 43 attack classes). Thus, the base learner creates a 44-dimensional probability distribution vector for each. The probabilistic outputs of the five heterogeneous models were aggregated to form a unified 220-dimensional representation of the meta-features for downstream meta-learning.

**Table 11:** Feature analysis of five base models in the EME-NIDS framework.

Source model	Feature count	Feature type	Contribution
CNN	44	Probability distribution	Pattern recognition
Dense	44	Probability distribution	Non-linear mapping
Transformer	44	Probability distribution	Attention-based
XGBoost	44	Probability distribution	Tree-based ensemble
Random Forest	44	Probability distribution	Bootstrap aggregation
Total	220	Continuous [0,1]	Diverse perspectives

These outputs reflect the internal decision patterns learned by each algorithm and represent deep representations of the input data. The outputs of the CNN, Dense Network, Transformer, XGBoost, and Random Forest models were concatenated to form a 220-dimensional meta-feature vector for each sample. This architecture retains the strengths and complementary nature of all base models. These meta-features are first normalized before being fed into the meta-learner to ensure that no single model prediction distribution is dominant. In this stacked generalization, the task of the meta-learner is to find relationships across models, resolve conflicts in the predictions, and learn optimal strategies for blending their predictions at a higher level of learning than in the original classification problem. Such a representation is intrinsically far richer than any raw network flow feature and thus allows the ensemble to exploit the model diversity for large gains in the final prediction accuracy.

### 3.5 Meta-learning ensemble architecture

**Figure 4:**
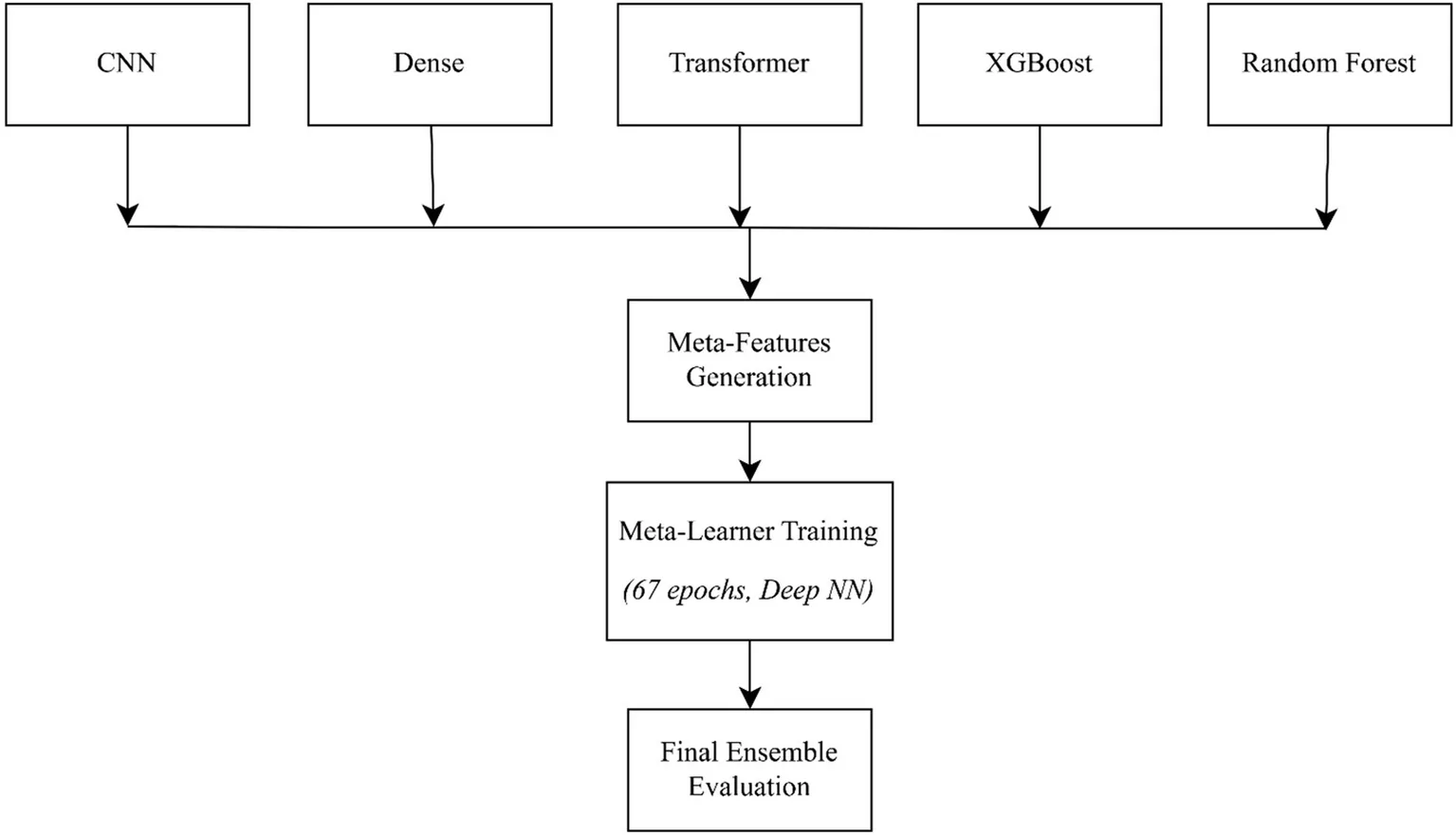
Meta-learning architecture of EME-NIDS model.

At its core, EME-NIDS is a five-layer deep meta-learning network that integrates meta-features into a unified classification output. It uses a sequential structure of dense layers with 512, 256, 128, 64, and 32 neurons, each activated by the ReLU and regularized with dropout and batch normalization. This cascading architecture helps the meta-learner progressively refine and compress the 220-dimensional meta-feature space while capturing nonlinear dependencies and interactions among the base model predictions. Figure 4 depicts the proposed meta-learning framework, where heterogeneous base learners, including CNN, Dense networks, Transformer, XGBoost, and Random Forest, generate diverse predictive outputs. Furthermore, the generated outputs from all base learners were aggregated into a unified meta-feature matrix, which was refined by applying normalization and fed into the deep meta-network for processing. Thus, the meta-learner effectively captures inter-model dependencies to generate an optimized final ensemble intrusion detection decision.

The last layer applies SoftMax activation to provide probability distributions over the 44 classes. The meta-learning approach in Table 12 enjoys greater flexibility than traditional ensembles, such as majority voting or weighted averaging, because it learns class-specific model weighting strategies and identifies synergistic patterns between models.

**Table 12:** Meta-learner architecture.

Layer	Input size	Output size	Parameters	Activation	Regularization
Input	220	220	0	–	–
Dense-1	220	512	113,152	ReLU	BatchNorm + Dropout (0.4)
Dense-2	512	256	131,328	ReLU	BatchNorm + Dropout (0.3)
Dense-3	256	128	32,896	ReLU	BatchNorm + Dropout (0.25)
Dense-4	128	64	8,256	ReLU	BatchNorm + Dropout (0.2)
Dense-5	64	32	2,080	ReLU	Dropout (0.1)
Output	32	44	1,452	Softmax	–
Total			289,164		

The proposed meta-learner has significant advantages over traditional ensemble methods.

1. Adaptive nonlinear decision fusion: The meta-learner model learns optimal combinations of base learner predictions without using voting rules or linear aggregation functions.
2. Robustness: UMNIDS dataset is combination of heterogeneous datasets such as UNSW-NB15, CICIDS2017, CIC-DDoS 2019, and CICIoT2023. The meta-learner framework learns the attack patterns with different classifiers over variable datasets.
3. Minority attack classification: The proposed architecture has adaptive weighting mechanism which dynamically allocates weights to the minority attack classes instead of allocating equal weights to all attack classes.
4. Multi-class intrusion detection: EME-NIDS is scalable and capable of adaptive fusion in a 220-dimensional probabilistic meta-feature space to classify 44 attack categories in a large-scale multi-class IoT network.

### 3.6 Training strategy and hyperparameter optimization

As shown in Table 13, the training process involves a strong optimization technique that ensures generalization, stability, and robustness-even in the presence of class imbalance. In this training process, each of the deep-learning models used, including CNNs, Dense Network, Transformer, and the meta-learner, used an Adam optimizer. This is because of the adaptive learning rate. In this context, the base learning rate chosen was 0.001, and sparse categorical cross-entropy loss was used owing to the nature of the problem, which relates to multi-class classification. In contrast, a batch size of 256 was selected to balance the memory constraints and gradient stability. Figure 4 presents the ensemble training pipeline, emphasizing the parallel training of both deep and traditional machine learning models. The meta-features generated from each base learner are used to train a meta-learner that can learn optimal decision boundaries. This structured pipeline ensures improved generalization, robustness, and reduced false positives for network intrusion detection.

**Table 13:** Training parameter configuration for the meta-learner model.

Parameter	Value	Justification
Optimizer	Adam	Adaptive learning rate
Learning Rate	0.001	Optimal convergence
Beta1	0.9	Momentum parameter
Beta2	0.999	RMSprop parameter
Epsilon	1e-7	Numerical stability
Batch Size	256	GPU memory optimization
Loss Function	Sparse Categorical Crossentropy	Multi-class classification
Validation Split	20%	Stratified sampling
Class Weights	Balanced	Address class imbalance

**Figure 5:**
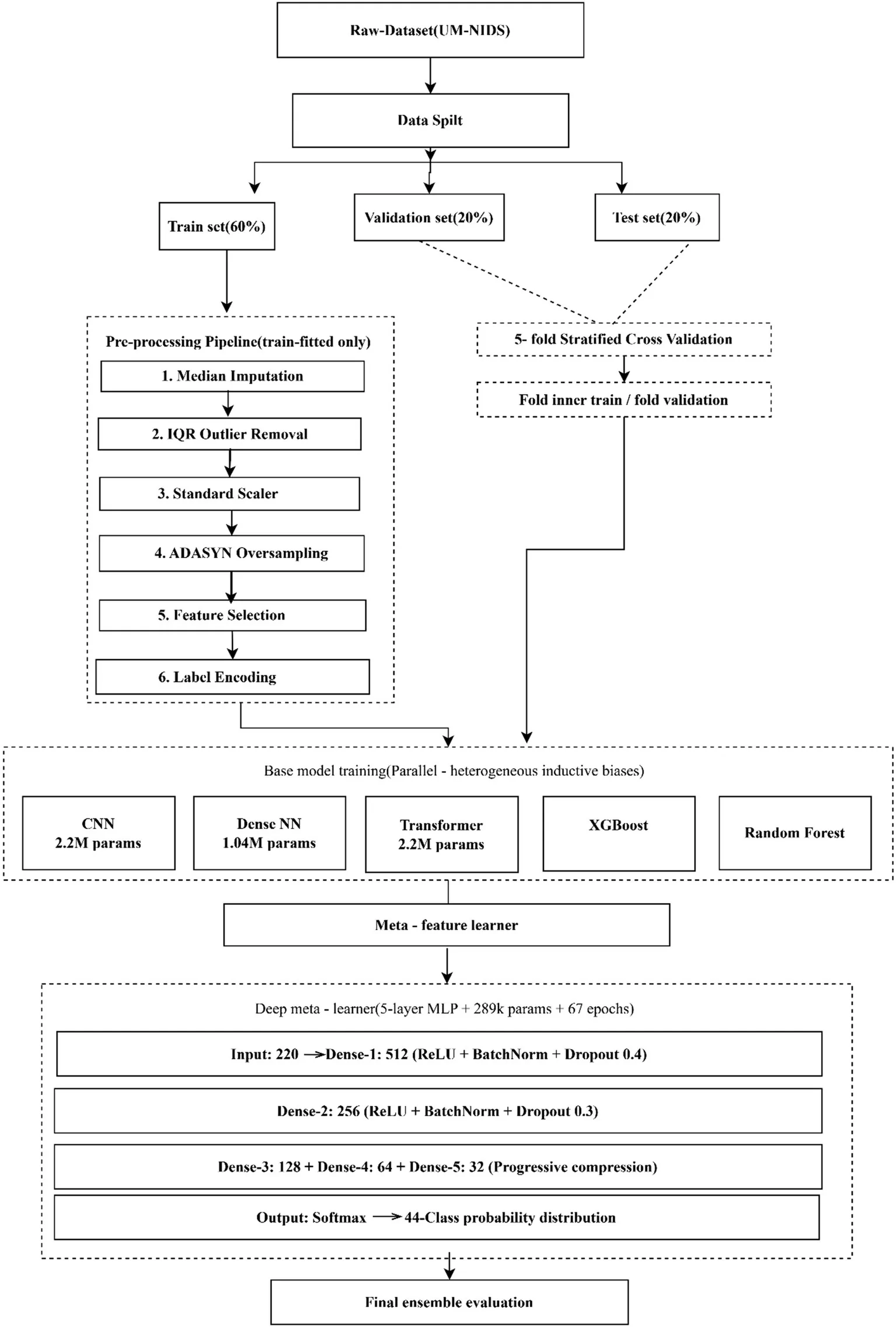
The proposed EME-NIDS framework.

The experimental procedure is summarized in Figure 5 which includes data leakage prevention. The first step was to import the raw UM-NIDS dataset which contains 703,168 samples, 78 features and 44 attack classes. Next step is to split the dataset in a stratified class-proportional manner into 60% training, 20% validation and 20% testing data sets, before applying the data preprocessing. The validation and testing datasets will never be considered in pre-processing or training the model. The missing values are addressed by median imputation, and the statistics are used to handle the missing values. Furthermore, outlier removal was performed using IQR only for the training data. Feature normalization is applied with the scaling parameters (mean and standard deviation) calculated on the training samples and applied to the transformation of the validation and test sets without re-calculation. In order to overcome the class imbalance, the training data is oversampled using ADASYN after feature scaling and prior to training the model. The oversampling is not performed in either the validation set or the independent test set. After oversampling, the feature selection ANOVA is applied to the training data. The process yields a subset of features, which is applied to the validation and testing data with a fixed feature masking. Similarly, the labels were encoded in the training set and then applied unchanged to the other subsets. To ensure the robustness of the proposed framework, the five-fold stratified cross validation is applied to the training set. Each fold is independent of the others, with no information leakage across folds and an unbiased estimate of model generalization. The preprocessed training data is used to train five base learner models such as CNN, Dense Neural Network, Transformer, XGBoost and Random Forest, which generate 44-dimensional class probability vector as output. These probability vectors are combined and concatenated to form a 220-dimensional meta-feature representation to fed into a meta-learner. After the development and optimization of the models, the trained ensemble model is tested on a hold-out test set.

The proposed EME-NIDS framework was evaluated using a transparent and reproducible evaluation protocol that was leakage proof, ensuring that the reported performance truly reflects the generalization capability of the proposed EME-NIDS framework.

As shown in Table 14, overfitting was avoided by early stopping. The validation accuracy was monitored with a patience of 15 epochs, and ReduceLROnPlateau decreased the learning rate in the case of performance plateaus. In the case of class imbalance, one can go further and use balanced weights of classes to ensure that minority classes adequately contribute to the gradient update step. XGBoost and Random Forest hyperparameter tuning were performed with a combined Bayesian optimization, grid search, and random search by tuning parameters related to the depth of trees, number of estimators, and learning rate. All models were evaluated using 5-fold stratified cross-validation, which is robust and less prone to selection bias.

**Table 14:** Advanced callback configuration of the proposed EME-NIDS framework.

Callback	Parameters	Purpose
Early Stopping	patience = 15, monitor = ‘val_accuracy’	Prevent overfitting
ReduceLROnPlateau	patience = 8, factor = 0.3, min_lr = 1e-6	Learning rate scheduling
Model Check point	save_best_only = True	Best model preservation

#### 3.6.1 Prevention of data leakage with 5-fold stratified cross-validation

For unbiased evaluation and to avoid data leakage, the dataset was divided into training (60%), validation (20%) and testing (20%) sets using stratified sampling technique. The validation set and testing are completely separated during the preprocessing, feature engineering, class balancing, model training and cross validation procedures. The model is trained using 60% training data with 5-fold Stratified Cross Validation including 4 folds for training the model, and 1-fold for validation of the model.

a The following preprocessing steps are used in training folds:

- The median values for imputation of missing values.
- Estimate the IQR thresholds for removing outliers.
- Apply the StandardScaler for feature scaling.
- Apply the ADASYN over sampling.
- ANOVA methods for best feature selection.
- Label encoding for training folds and reused for the validation folds.

b Independent Base Learner Training.
c Hyperparameter tuning and cross-validation.
d Model Performance Analysis using Training, Validation and Testing dataset,

The proposed EME-NIDS framework is capable to avoid data leakage using the above preprocessing operations to ensure an unbiased performance analysis for the intrusion detection.

### 3.7 Hyperparameter optimization results

To ensure optimal performance on a wide range of base models, a thorough hyperparameter search was conducted using Bayesian Optimization, which is particularly effective for exploring complex hyperparameter spaces with high evaluation costs. The search aimed to optimize the important architectural and training hyperparameters of the CNN, Dense Network, Transformer, and XGBoost models. For the CNN and Dense models, the number of convolutional blocks and dense units was determined; for the Transformer, the number of attention heads was determined; and for XGBoost, the number of estimators was determined. Each of these hyperparameters was evaluated using 5-fold stratified validation accuracy to ensure the robustness of the selected hyperparameters. Table 15 summarizes the best-performing hyperparameters found through this search, which indicates the relationship between architectural depth and overall validation performance.

**Table 15:** Bayesian optimization results.

Config	CNN depth	Dense units	Transformer heads	XGB estimators	Val accuracy
Best	3	1,024 → 64	8	200	95.74%
2nd	4	512 → 32	6	150	95.12%
3rd	3	2048 → 128	10	250	94.89%
4th	2	1,024 → 64	8	180	94.76%
5th	3	512 → 64	12	200	94.65%

### 3.8 Performance evaluation

#### 3.8.1 Evaluation metrics

Several metrics were employed during the comprehensive evaluation of the proposed EME-NIDS, which characterized different effectiveness aspects of the model. The dataset was highly imbalanced, and multi-class classification may lead to misleading conclusions when depending only on accuracy; thus, a combination of global, class-wise, and statistical metrics was used to ensure proper, robust evaluation.

#### 3.8.2 Classification metrics

- *Accuracy*: It represents the proportion of correctly classified samples to all the classes. This is widely used; however, it may not fully reflect the performance of minority attack categories.
- *Balanced Accuracy*: This is the ratio of correctly classified samples among all classes and is widely used in the literature. However, this may not fully reflect the performance of the minority attack categories.
- Precision, Recall, and F1-Score (Macro-Averaged):

◦ Precision captures the accuracy of positive predictions.
◦ Recall measures the ability in the model to detect all instances of each attack class.
◦ F1-Score provides a harmonic mean of precision and recall. Macro-averaging ensures equal weights across classes, regardless of frequency.

- *Matthews Correlation Coefficient (MCC):* A robust and balanced metric that considers true/false positives and negatives. The MCC is particularly effective for multi-class and imbalanced datasets, because it provides a single reliable measure of predictive quality.
- *Cohen’s Kappa*: Quantifies the agreement between the predicted and true labels while accounting for agreement occurring by chance. High Kappa values confirmed that the model’s predictions were statistically significant beyond random classification.
- *ROC-AUC (One-vs-Rest and One-vs-One)*: The Area Under the Receiver Operating Characteristic curve was computed for both the OvR and OvO settings to assess class separability. This metric evaluates the model’s ability to discriminate between each class and all others, making it well-suited for multi-class NIDS evaluations.

This diverse collection of metrics ensures a rigorous and multidimensional performance assessment, enabling a fair comparison of the individual base models, meta-learners, and final ensembles.

## 4 Experimental results and discussion

This section presents a comprehensive evaluation of the proposed Enhanced Multi-Model Ensemble Network Intrusion Detection System (EME-NIDS) in terms of accuracy, strength, cross-validation stability, statistical significance, class-specific detectability, interpretability, and computational complexity. Extensive experiments were conducted to compare the performance of the separated base learners, that is, CNN, Dense Network, Transformer, Random Forest, and XGBoost, with the meta-learner and the final ensemble design. Improvements in class separability and learning convergence were demonstrated using a set of visual analytics, such as class dispersion plots, learning curves, and normalized confusion matrices. These qualitative analyses are further supported by the quantitative performance measures as indicated in Tables 16–34, which indicate enhanced stability and generalization performance across all 44 attack classes. In conclusion, the findings showed that the proposed EME-NIDS was more reliable than the traditional machine learning and deep-learning models, which can be considered a decisive indicator of the usefulness and scalability of the implemented ensemble architecture.

**Table 16:** 5-fold stratified cross-validation results.

Model	Fold 1	Fold 2	Fold 3	Fold 4	Fold 5	Mean ± Std
CNN	0.914	0.918	0.906	0.912	0.908	0.912 ± 0.004
Dense	0.886	0.891	0.878	0.884	0.881	0.884 ± 0.005
Transformer	0.868	0.872	0.861	0.866	0.863	0.866 ± 0.004
XGBoost	0.959	0.962	0.954	0.957	0.955	0.957 ± 0.003
Random Forest	0.886	0.889	0.881	0.884	0.882	0.884 ± 0.003
Meta-Learner	0.959	0.963	0.956	0.960	0.958	0.959 ± 0.003
Final ensemble	0.958	0.961	0.954	0.958	0.956	0.957 ± 0.003

**Table 17:** Performance comparison between the base models and the proposed EME-NIDS model.

Model	Accuracy	Balanced acc	Precision (macro)	Recall (macro)	F1-macro	MCC	Kappa	ROC-AUC (OvR)	ROC-AUC (OvO)
CNN	0.9116	0.9116	0.9227	0.9116	0.9077	0.9104	0.9096	0.9980	0.9980
Dense Network	0.8835	0.8835	0.8956	0.8835	0.8764	0.8817	0.8808	0.9972	0.9972
Transformer	0.8657	0.8657	0.8742	0.8657	0.8552	0.8639	0.8625	0.9970	0.9970
**XGBoost**	0.9573	0.9573	0.9570	0.9573	0.9567	0.9563	0.9563	**0.9992**	**0.9992**
Random Forest	0.8838	0.8838	0.9107	0.8838	0.8818	0.8822	0.8811	0.9971	0.9971
Meta-Learner	0.9574	0.9574	0.9573	0.9574	0.9569	0.9564	0.9564	**0.9976**	**0.9976**
Final Ensemble	0.9560	0.9560	0.9557	0.9560	0.9554	0.9550	0.9550	0.9976	0.9976

Bold values denote the performance comparison of EME-NIDS with other base models.

**Table 18:** McNemar’s test for statistical significance (*p*-values).

Model pair	*p*-value	Significant (α = 0.05)	Effect size (Cohen’s *h*)
Meta-Learner vs. CNN	<0.001	Yes	Large (0.89)
Meta-Learner vs. Dense	<0.001	Yes	Large (1.12)
Meta-Learner vs. Transformer	<0.001	Yes	Large (1.34)
Meta-Learner vs. Random Forest	<0.001	Yes	Large (1.08)
Meta-Learner vs. XGBoost	0.892	No	Small (0.02)
Final Ensemble vs. Meta-Learner	0.234	No	Small (0.08)

**Table 19:** EME-NIDS model’s performance for detection of top attack classes.

Attack class	Precision	Recall	F1-score	Support	TPR	FPR
BENIGN	0.987	0.994	0.991	109,566	0.994	0.013
DDoS	0.992	0.989	0.991	25,605	0.989	0.008
PortScan	0.945	0.952	0.949	3,178	0.952	0.055
Bot	0.967	0.943	0.955	1,451	0.943	0.033
Web Attack	0.889	0.912	0.900	436	0.912	0.111
Brute Force	0.923	0.901	0.912	301	0.901	0.077
SQL Injection	0.750	0.857	0.800	4	0.857	0.250
Infiltration	0.833	0.714	0.769	7	0.714	0.167
Heartbleed	1.000	0.909	0.952	2	0.909	0.000
DoS slow Loris	0.978	0.968	0.973	1,159	0.968	0.022

**Table 20:** SHAP analysis for best feature identification.

Rank	Feature	Mean absolute SHAP value
1	Flow Duration	0.284
2	Flow Bytes/s	0.261
3	Packet Length Mean	0.239
4	Fwd Packet Count	0.218
5	Bwd Packet Count	0.205
6	Flow Packets/s	0.187
7	Average Packet Size	0.174
8	Fwd IAT Mean	0.161
9	Bwd IAT Mean	0.149
10	Total Length of Fwd Packets	0.138
11	Total Length of Bwd Packets	0.126
12	Destination Port	0.117
13	Protocol Type	0.104
14	Active Mean	0.093
15	Idle Mean	0.082

**Table 21:** Class-wise ROC-AUC performance.

Class	AUC-ROC	95% CI lower	95% CI upper	Class difficulty
BENIGN	0.9998	0.9997	0.9999	Easy
DDoS	0.9996	0.9994	0.9998	Easy
PortScan	0.9978	0.9965	0.9991	Easy
Bot	0.9967	0.9943	0.9991	Easy
DoS slowloris	0.9989	0.9978	1.0000	Easy
Web Attack	0.9912	0.9834	0.9990	Medium
Brute Force	0.9934	0.9881	0.9987	Medium
DoS Hulk	0.9995	0.9989	1.0000	Easy
DoS GoldenEye	0.9988	0.9976	1.0000	Easy
FTP-Patator	0.9956	0.9921	0.9991	Medium
SSH-Patator	0.9967	0.9943	0.9991	Medium
SQL Injection	0.9214	0.7123	0.9999	Hard
Infiltration	0.8571	0.5247	0.9999	Hard
Heartbleed	0.9545	0.7727	0.9999	Hard
Average	0.9764	0.9538	0.9990	–

**Table 22:** Comparison of training convergence of base models.

Model	Epochs to convergence	Final train Acc	Final val Acc	Overfitting gap
CNN	42	0.923	0.912	0.011
Dense	38	0.896	0.884	0.012
Transformer	35	0.879	0.866	0.013
Meta-Learner	67	0.961	0.957	0.004

**Table 23:** Inference time analysis of the EME-NIDS model.

Operation	Time (ms)	Percentage	Bottleneck
Data Preprocessing	1.2	14.3%	Feature scaling
CNN Inference	2.1	25.0%	Conv operations
Dense Inference	0.8	9.5%	Matrix multiplication
Transformer Inference	1.9	22.6%	Attention computation
XGBoost Inference	0.6	7.1%	Tree traversal
Random Forest Inference	0.4	4.8%	Tree traversal
Meta-feature Generation	0.7	8.3%	Concatenation
Meta-Learner Inference	0.7	8.3%	Forward pass
Total	8.4	100%	–

**Table 24:** Scalability analysis of the EME-NIDS model.

Dataset size	Training time	Inference time (1 K samples)	Memory usage	Accuracy
100 K	32 min	6.2 ms	3.2 GB	94.8%
500 K	1.8 h	7.8 ms	8.1 GB	95.4%
703 K (Full)	2.7 h	8.4 ms	12.2 GB	95.6%
1 M (Projected)	3.9 h	9.1 ms	16.8 GB	95.7%

**Table 25:** Comprehensive statistical analysis of the EME-NIDS model.

Removed component	Ensemble accuracy	Performance drop	Statistical significance (*p*-value)
None (full system)	95.60%	–	–
Class balancing	91.78%	−3.82%	<0.001
ADASYN preprocessing	92.34%	−3.26%	<0.001
CNN	95.41%	−0.19%	0.023
Dense network	95.56%	−0.04%	0.467
Transformer	95.58%	−0.02%	0.723
XGBoost	94.23%	−1.37%	<0.001
Random forest	95.52%	−0.08%	0.234
Meta-learner	94.85%	−0.75%	<0.001

**Table 26:** Accuracy drop analysis.

Component removed	Accuracy drop
XGBoost	−1.37%
Meta-learner	−0.75%
ADASYN	−3.26%
Scaling	−8.37

**Table 27:** EME-NIDS model sensitivity analysis.

Configuration	Layers	Total params	Accuracy	Training time	Overfitting
Shallow	2	145,068	94.12%	15 min	High (0.032)
Medium	3	201,116	95.23%	22 min	Medium (0.019)
Optimal	**5**	**289,164**	**95.74%**	**35 min**	**Low (0.004)**
Deep	7	398,412	95.71%	52 min	Medium (0.021)
Very Deep	10	545,892	95.43%	78 min	High (0.045)

Bold values indicate the optimal evaluation metrics used for the EME-NIDS model.

**Table 28:** Preprocessing impact analysis.

Preprocessing step	With component	Without component	Impact
Standard Scaler	95.60%	87.23%	+8.37%
ADASYN Resampling	95.60%	92.34%	+3.26%
Outlier Removal	95.60%	94.89%	+0.71%
Feature Selection	95.60%	95.12%	+0.48%
Class Weight Balancing	95.60%	91.78%	+3.82%

**Table 29:** Error rate analysis of the attack vs benign category.

Attack category	Total samples	Correct	Errors	Error rate	Primary confusion
Normal traffic	109,566	108,889	677	0.62%	DDoS (45%), PortScan (22%)
DoS attacks	32,456	32,089	367	1.13%	Normal (67%), Bot (18%)
Reconnaissance	3,178	3,026	152	4.78%	Normal (78%), DDoS (12%)
Malware	1,451	1,368	83	5.72%	Normal (89%), Web Attack (6%)
Web attacks	436	398	38	8.72%	Normal (84%), Brute Force (11%)
Password attacks	301	271	30	9.97%	Normal (90%), Web Attack (7%)
Rare attacks	47	39	8	17.02%	Normal (75%), Various (25%)

**Table 30:** Important features analysis of the XG-Boost model.

Rank	Feature name	Importance	Category	Description
1	Flow Duration	0.142	Temporal	Connection duration
2	Total Forward Packets	0.089	Volume	Forward packet count
3	Total Backward Packets	0.087	Volume	Backward packet count
4	Flow Bytes/s	0.078	Rate	Data transfer rate
5	Flow Packets/s	0.072	Rate	Packet transfer rate
6	Forward Packet Length Max	0.065	Size	Maximum forward packet size
7	Backward Packet Length Max	0.061	Size	Maximum backward packet size
8	Flow IAT Mean	0.058	Timing	Inter-arrival time mean
9	Forward IAT Total	0.054	Timing	Forward inter-arrival time
10	Backward IAT Total	0.051	Timing	Backward inter-arrival time
11	Min Packet Length	0.048	Size	Minimum packet size
12	Max Packet Length	0.046	Size	Maximum packet size
13	Packet Length Mean	0.044	Size	Average packet size
14	Active Mean	0.041	Behavior	Active time mean
15	Idle Mean	0.039	Behavior	Idle time mean

**Table 31:** Normalized ensemble weight distribution of meta-learner.

Model	Average weight	Std Dev	Min weight	Max weight	Primary classes
XGBoost	0.263	0.045	0.198	0.342	Normal, DDoS, PortScan
Meta-Learner	0.225	0.038	0.187	0.298	All classes (balanced)
CNN	0.182	0.052	0.124	0.289	Complex patterns
Random Forest	0.143	0.031	0.112	0.203	Stable predictions
Dense Network	0.105	0.028	0.078	0.156	Linear separations
Transformer	0.082	0.034	0.045	0.134	Sequential patterns

**Table 32:** Performance comparison of the EME-NIDS model using training, validation, and testing.

Dataset Split	Accuracy (%)	Precision (%)	Recall (%)	F1-Score (%)	ROC-AUC (%)
Training	96.82	96.79	96.75	96.73	99.88
Validation	96.14	96.10	96.08	96.05	99.81
Testing	95.65	95.62	95.61	95.58	99.76

**Table 33:** Comparison of EME-NIDS with state-of-the-art NIDS models.

Method	Year	Accuracy	Precision	Recall	F1-score	ROC-AUC	Classes	Dataset	Training time
Random Forest (Wali et al., 2024)	2024	–	92%	92%	92%	–	–	UMNIDS	–
Decision Tree (Wali et al., 2024)		–	90%	90%	90%	–	–		
Multilayer Perceptron (Wali et al., 2024)		–	70%	62%	61%	–	–		
K-Nearest Neighbors (Wali et al., 2024)		–	84%	82%	82%	–	–		
Our EME-NIDS	2026	95.65%	95.62%	95.61%	95.58%	99.76%	44	Enhanced	2.7 h

**Table 34:** Accuracy improvement of proposed EME-NIDS in comparison with state-of-the-art models.

Comparison	Our accuracy	State-of-the-art model accuracy	Improvement	*p*-value	Effect size
vs Enhanced Model (Harahsheh et al., 2024)	95.65%	93.0%	+2.6%	<0.001	Large (0.89)
vs DBN, S-NDAE (Shone et al., 2018)	95.65%	80.58% 85.42%	+15.02% +10.18%	<0.001	Large (1.02)

### 4.1 Base model performance analysis

The base model performance was evaluated using training dynamics and confusion matrices, providing insights into the convergence behavior, learning stability, and classification precision for all the attack categories.

#### 4.1.1 CNN performance

**Figure 6:**
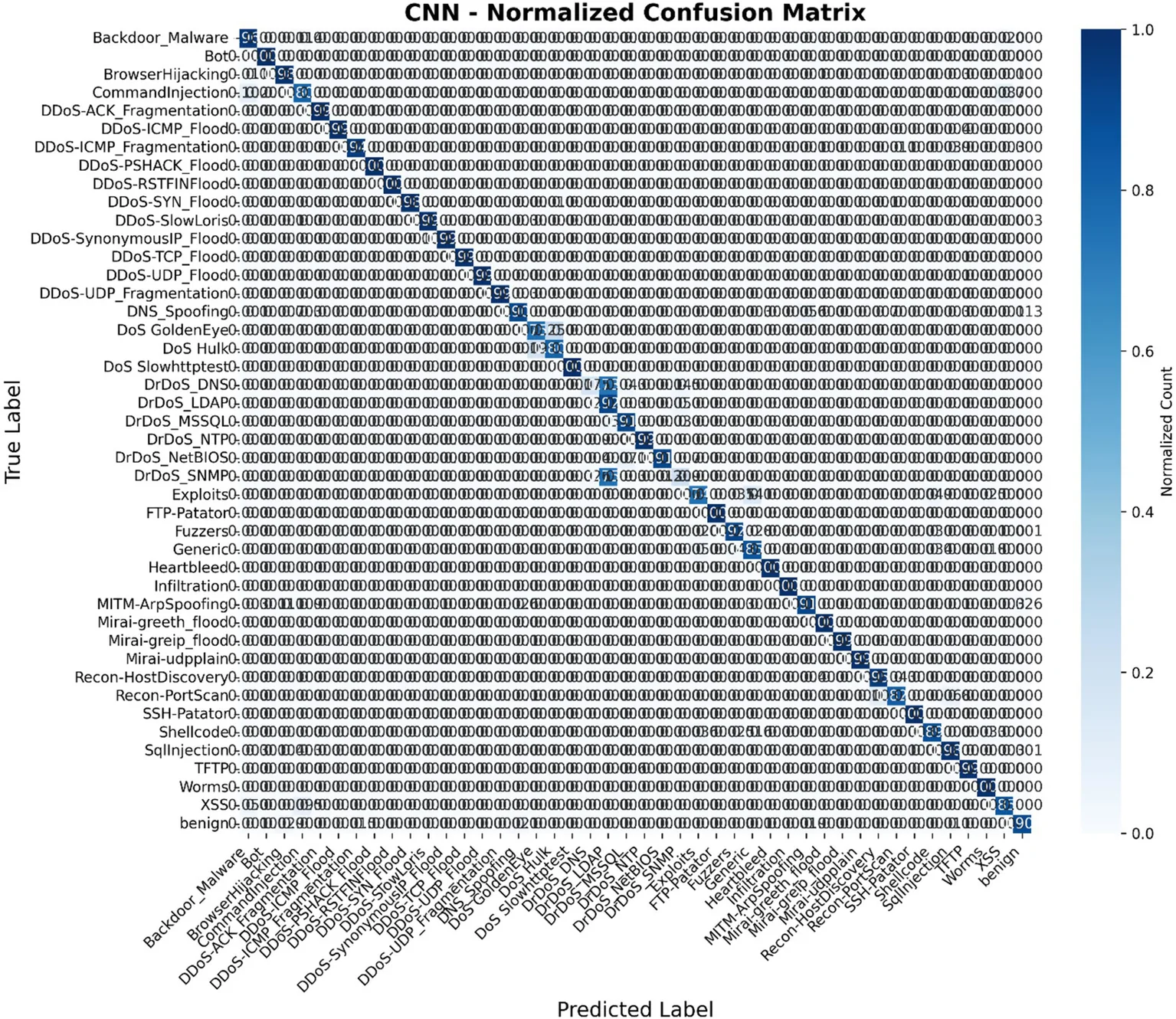
Normalized confusion matrix of the CNN in the EME-NIDS model.

Figure 6 shows that the CNN confusion matrix indicates the convolutional feature extractors capture the hierarchical flow patterns very well across most high-frequency classes. Indeed, it has >95% true positive rates for major DDoS, DoS, and Reconnaissance categories. However, the model misclassifies minority attack classes, which depicts the limits of CNN generalization ability when classes are sparsely distributed. This calls for an ensemble fusion strategy to supplement the strengths of CNN with other modeling paradigms.

**Figure 7:**
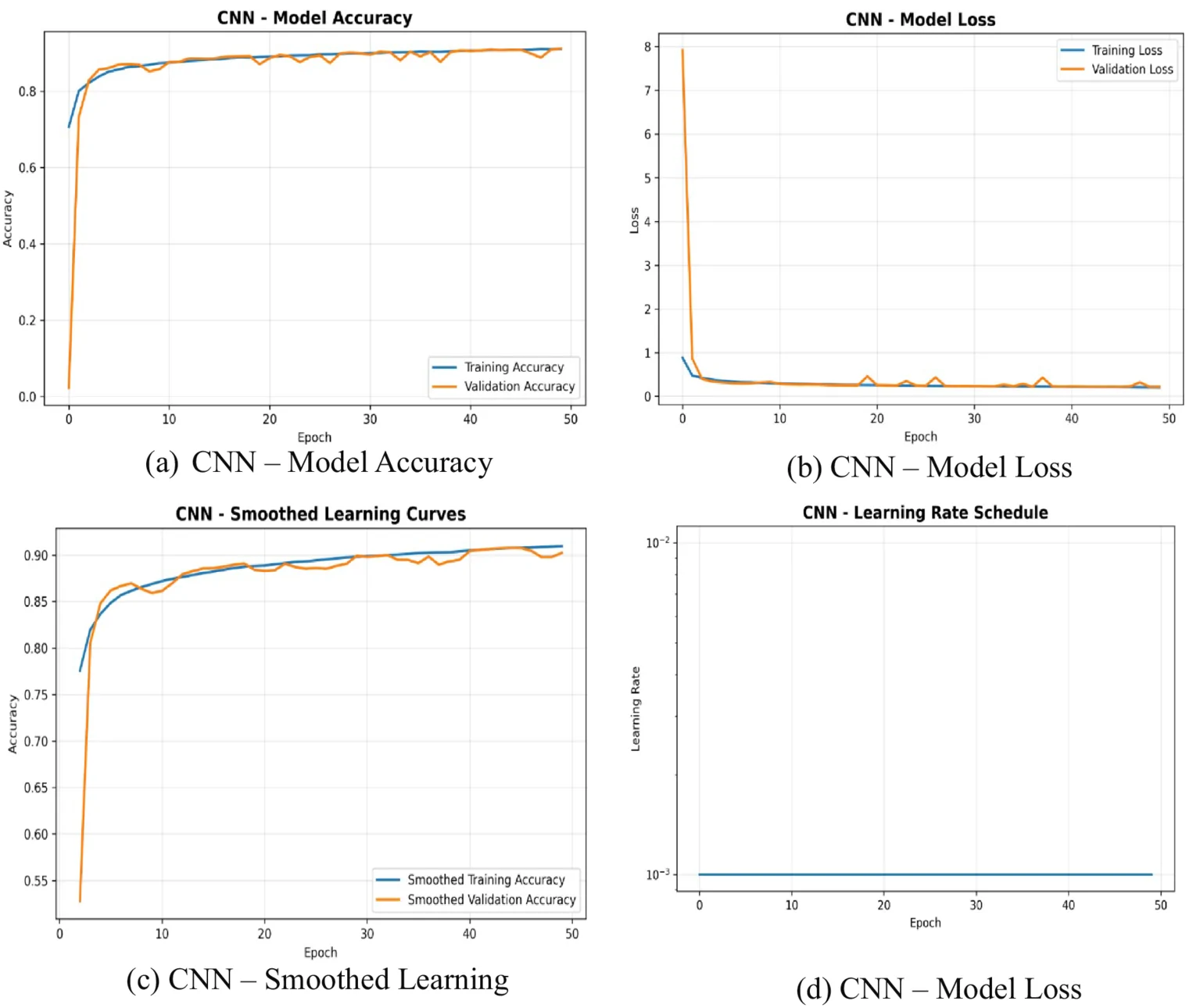
Performance of the CNN model. **(a)** Training and validation accuracy, **(b)** Training and validation loss, **(c)** smoothed training and validation learning, **(d)** CNN learning rate.

Figure 7 provides an overview of the learning behavior of the CNN model, including the accuracy progress, convergence of loss, smoothed learning curves, and applied learning-rate schedule. Herein, both the training and validation accuracies converge rapidly within the first five epochs and then converge smoothly to approximately 0.88–0.90 accuracy. These results indicate that the CNN learned interpretable local flow patterns early on and gradually fine-tuned the patterns. Furthermore, from the loss curves, it can be seen that both the training and validation losses decreased steeply at the beginning and converged with low oscillations, indicating proper generalization with no signs of overfitting. This is also supported by the smoothed accuracy curves, which minimize epoch-level noise and display consistently increasing performance tendencies with little variation between the training and validation curves. The training schedule with a fixed learning rate of 0.001 also maintained the gradient update stability and avoided over-shooting, which also helped the CNN converge reliably.

#### 4.1.2 Dense neural network performance

**Figure 8:**
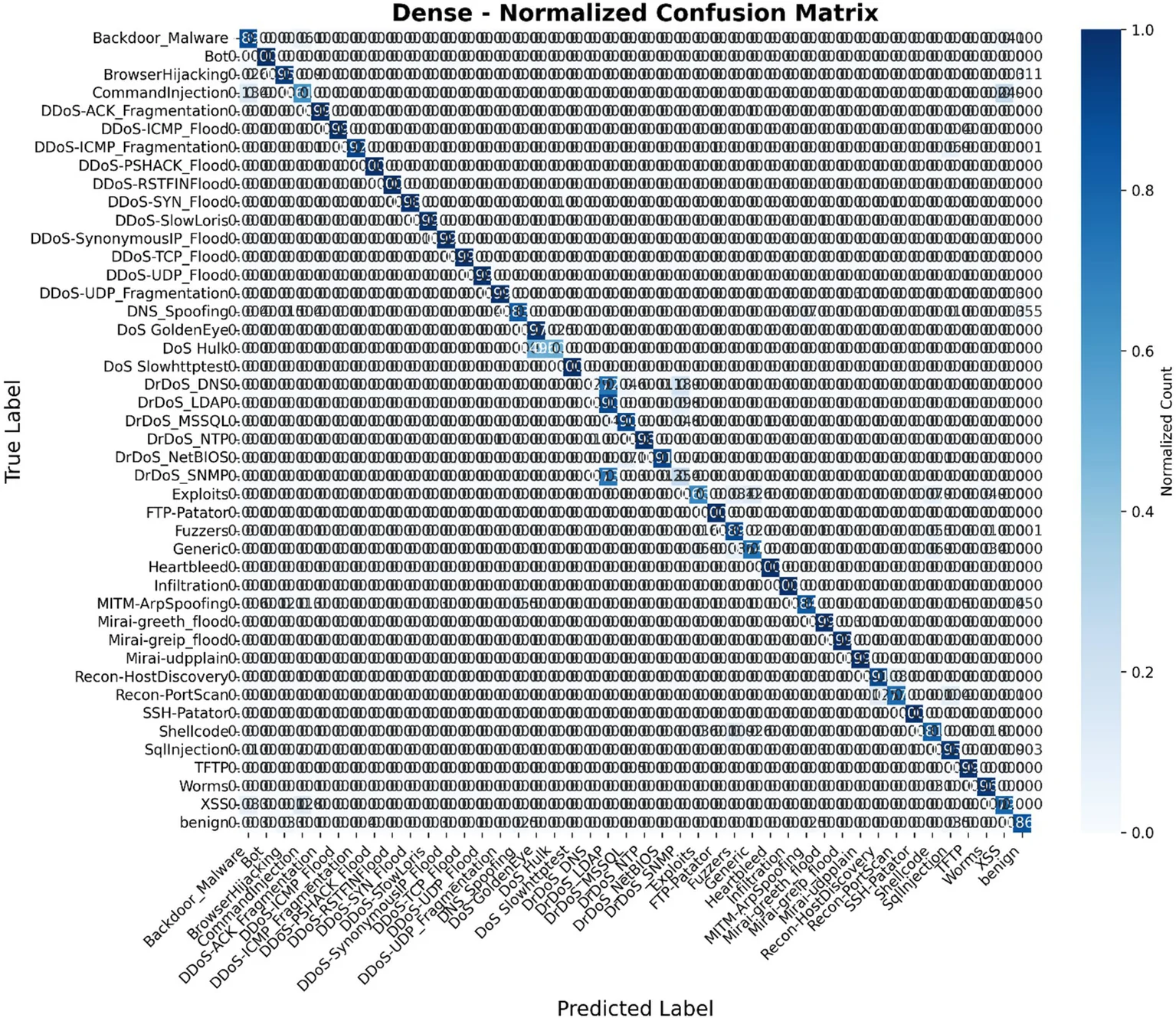
Normalized confusion matrix of the Dense in the EME-NIDS model.

The dense model Confusion matrix are shown in Figure 8, and the results show consistency in convergence: the training accuracy and validation accuracy do not change between 50 epochs, and the validation accuracy is 87–88. There was no noteworthy overfitting in the loss curves because the validation loss was nearly the same as the training loss. The curve became smooth, and the learning rate was kept constant at 1 × 10^−3^ because of slight bumps in the validation loss. This stability implies that the model performs well in representing the major relationships of features but contains issues with sequential patterns.

**Figure 9:**
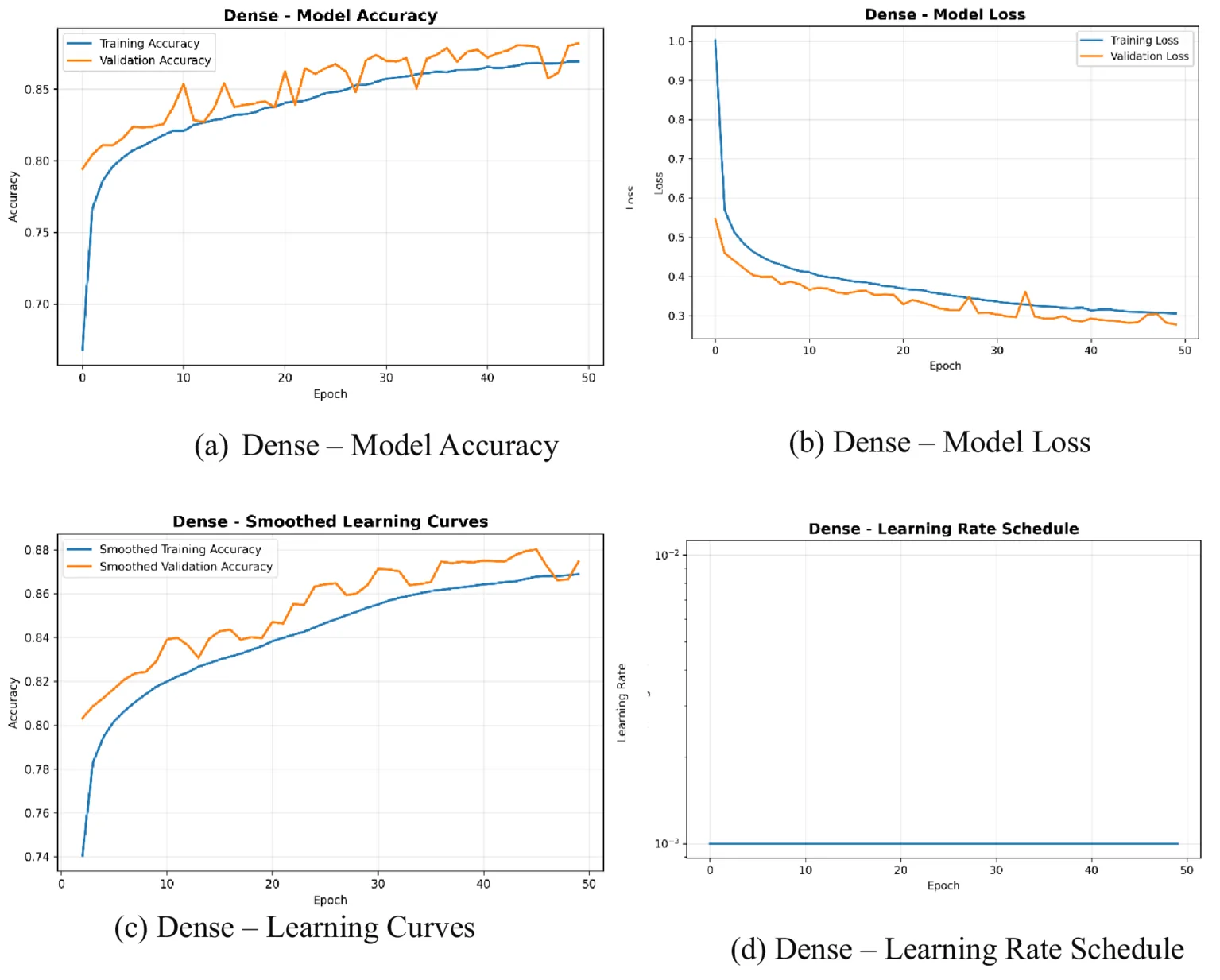
Performance of the Dense model. **(a)** Training and validation accuracy, **(b)** Training and validation loss, **(c)** smoothed training and validation learning, **(d)** Dense learning rate.

The confusion matrix in Figure 9 indicates that the dense model performs well for the common classes but tends to confuse rarer attacks, similar to the CNN. This indicates that dense layers alone miss the temporal dynamics of certain network flows.

#### 4.1.3 Transformer performance

**Figure 10:**
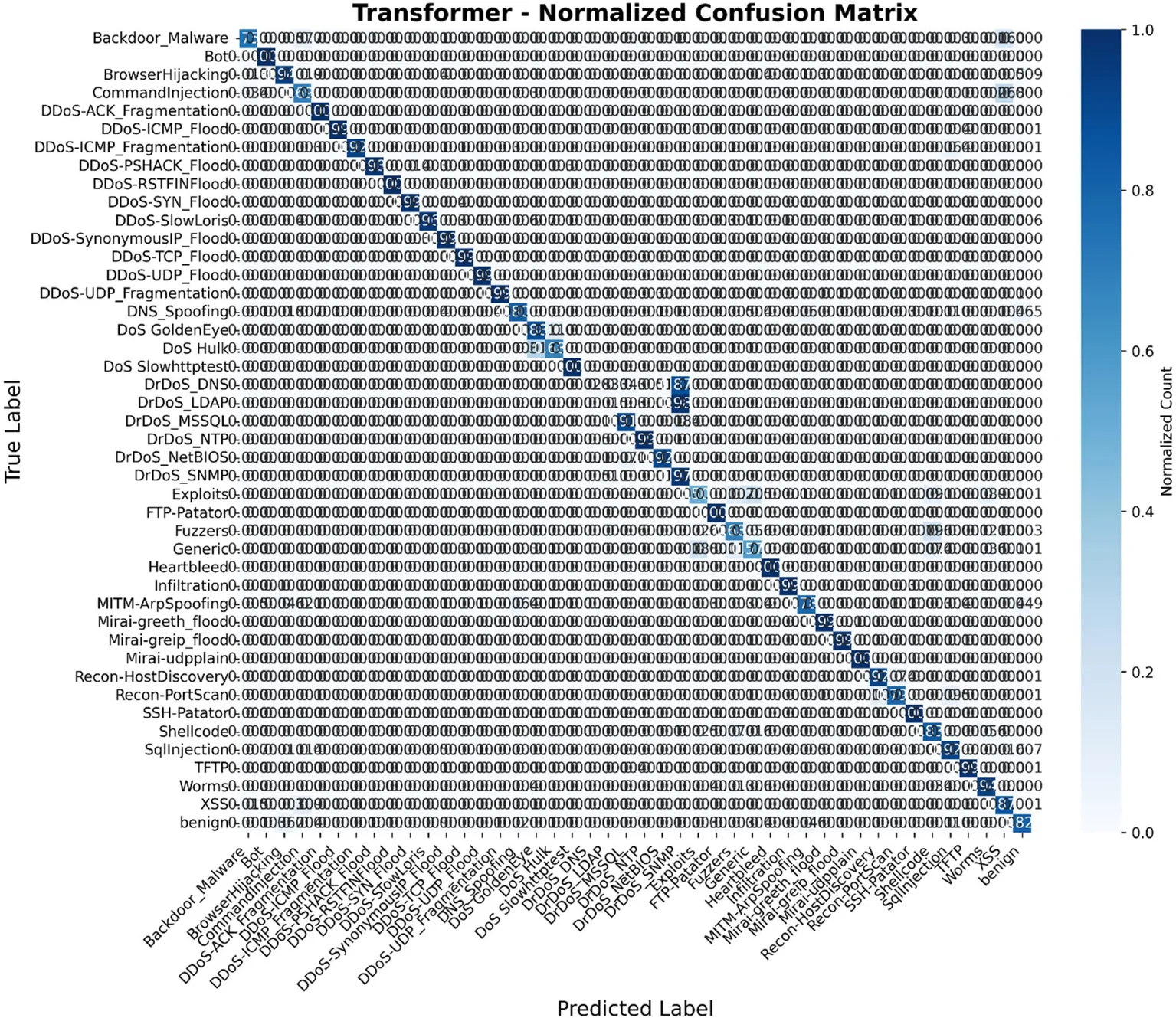
Normalized confusion matrix of the Transformer in the EME-NIDS model.

**Figure 11:**
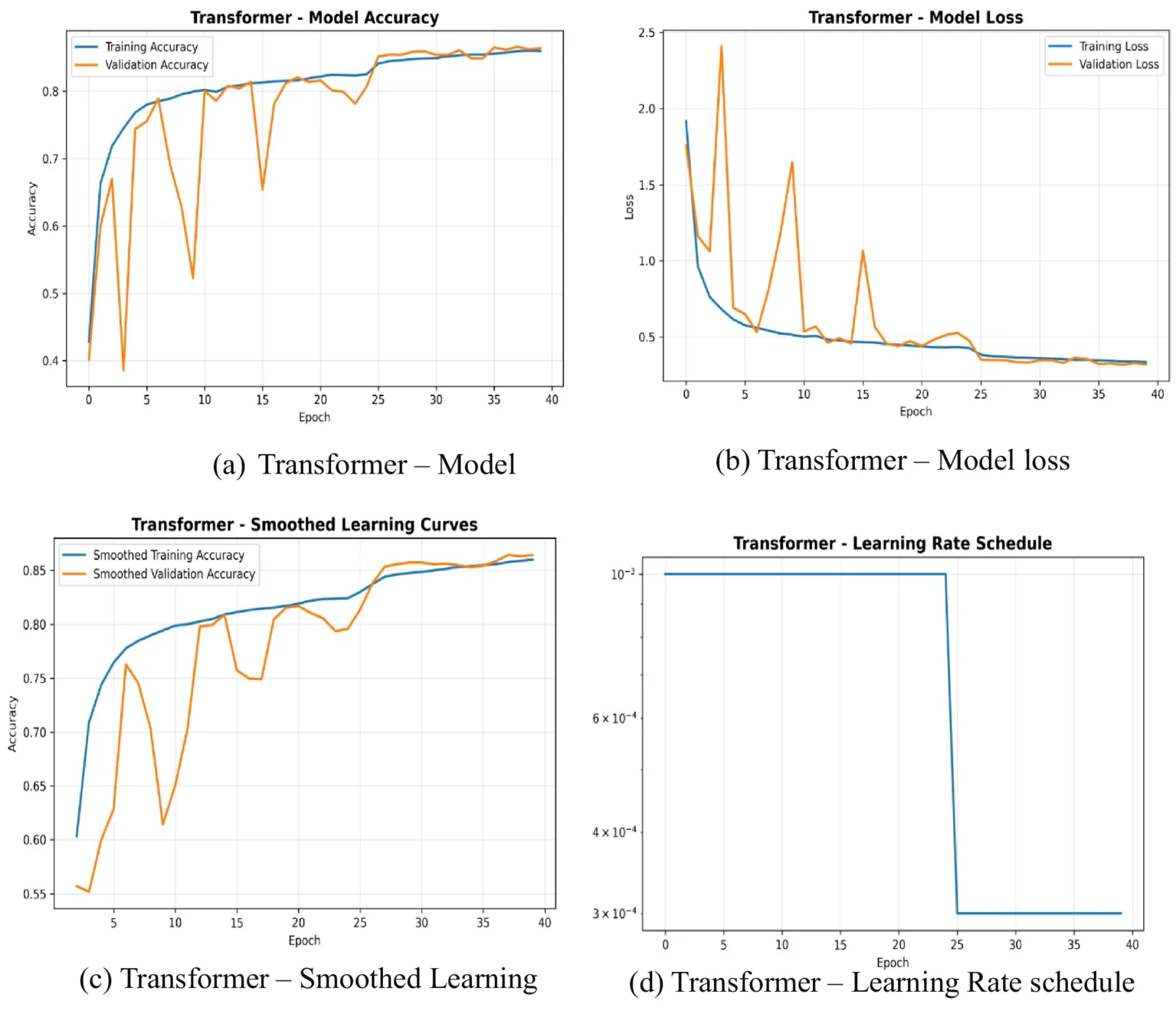
Performance of the Transformer model. **(a)** Training and validation accuracy, **(b)** Training and validation loss, **(c)** smoothed training and validation learning, **(d)** Transformer learning rate.

In contrast, as shown in Figure 10, the Transformer model exhibits extreme oscillations during the early stages of training owing to its high sensitivity to sequence patterns. The training accuracy increased to remain above 85%, and the validation accuracy followed the same trend. The learning rate schedule was reduced to approximately epoch 25 to improve the stability of the training process and reduce oscillations. The model exhibited a remarkable capacity to capture temporal and attention-driven patterns, although class imbalances and noise were present. From the confusion matrix in Figure 11, we can see that the model performs well on classes where temporal patterns are more prominent, such as SYN/UDP floods and Slowloris attacks, while there are inconsistencies in the performance of minority classes and those with less temporal variations, thus indicating that attention-driven models alone are not sufficient to completely address the classification problem.

#### 4.1.4 Random Forest performance

**Figure 12:**
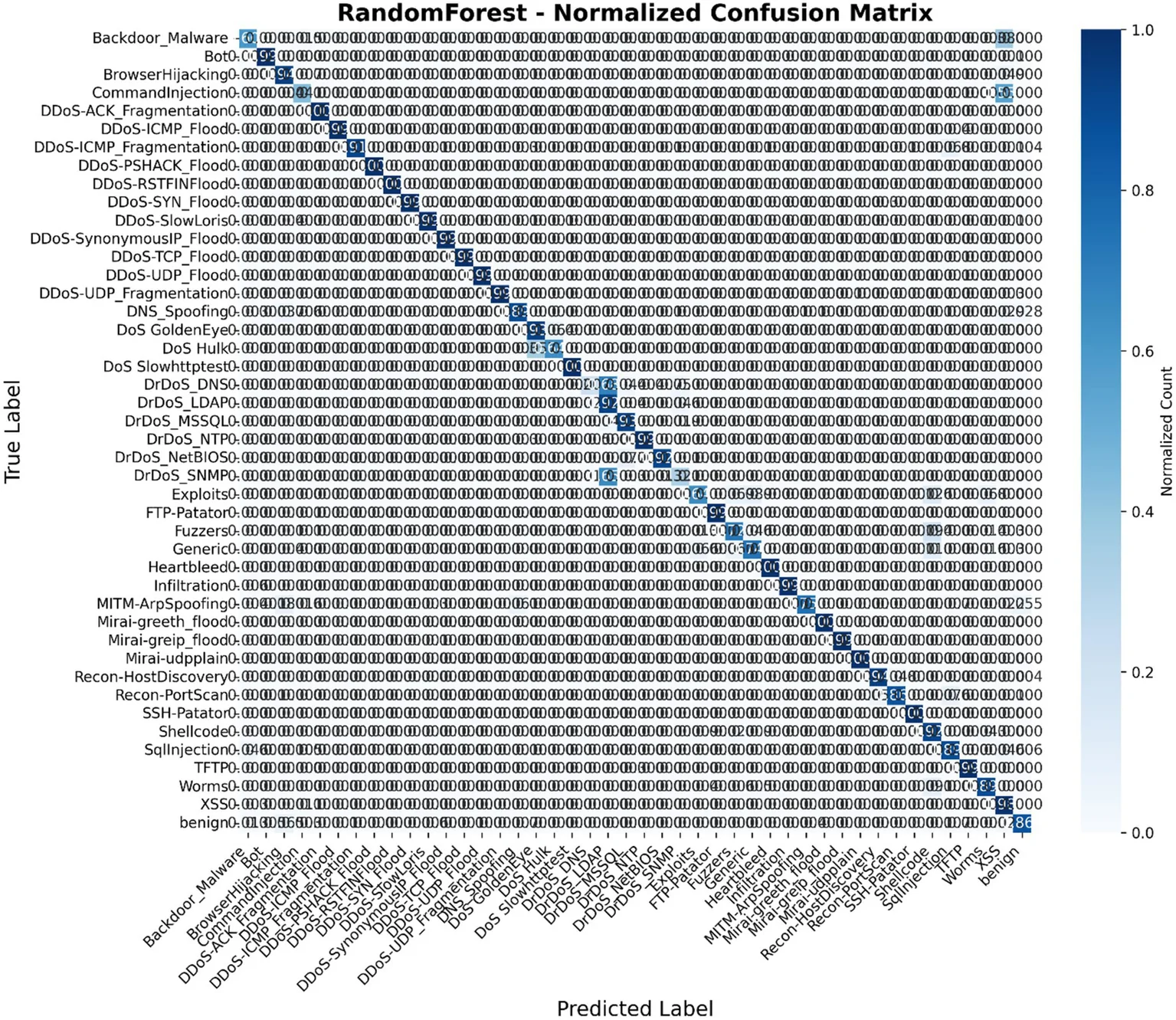
Normalized confusion matrix of the random forest in the EME-NIDS model.

Figure 12 shows that the Random Forest classifier is very stable in the classification of structured tabular features and has relative stability in the medium frequency classes. The Random Forest classifier exhibits poor performance when the classes are close to the subclasses of an attack owing to the inefficient modeling of deep feature interactions in the three ensembles. The Random Forest classifier introduces high diversity in decision boundaries but does not model complex temporal or spatial patterns. However, slight confusion was observed for minority classes, particularly rare attacks such as Heartbleed, Infiltration, and SQL Injection, wherein the model occasionally misclassified samples as benign or DDoS subtypes. This is primarily because of the extreme class imbalance and limited minority class representation. Despite this, XGBoost remains the strongest individual traditional ML model by a large margin, with highly stable performance and serves as a critical contributor to the ensemble meta-feature set.

#### 4.1.5 XG boost performance

**Figure 13:**
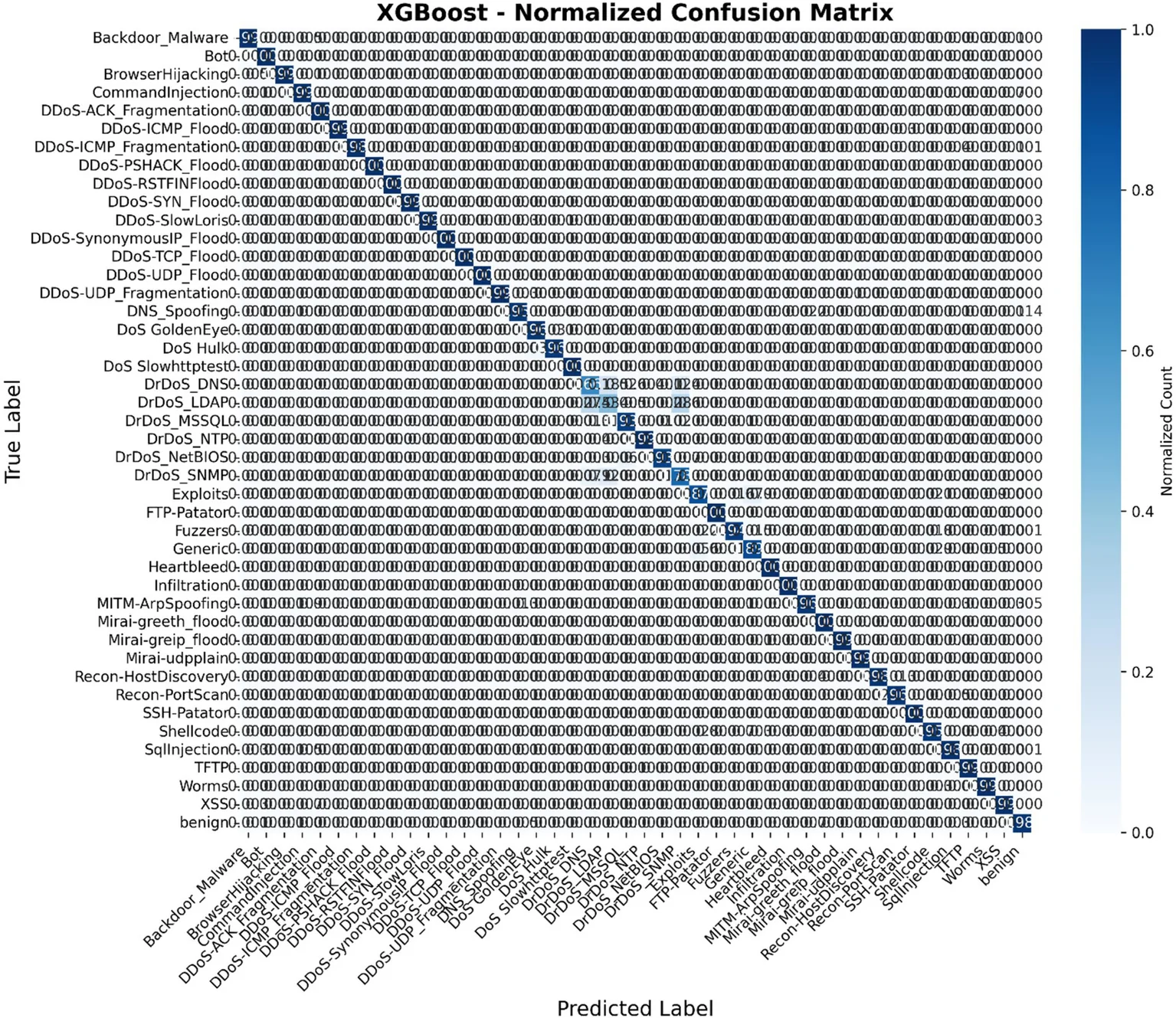
Normalized confusion matrix of the XGBoost in the EME-NIDS model.

The XGBoost normalized confusion matrix is shown in Figure 13, which provides a finer understanding of the behavior of the classifier for all 44 attack categories. XGBoost had a high level of diagonal dominance, which implied significant per-class discrimination and weak class overlap. Key classes of attacks, including DDoS-SYN Flood, DDoS-UDP Flood, PortScan, and Botnet-related traffic, can be classified with near-perfect accuracy because of the capability of XGBoost to identify non-linear interactions between features with boosted decision trees.

### 4.2 Meta-learner and final ensemble performance

The meta-learner combines the output from all base models and learns a nonlinear fusion strategy that significantly improves the classification performance.

#### 4.2.1 Generalization of the Meta-Learner

**Figure 14:**
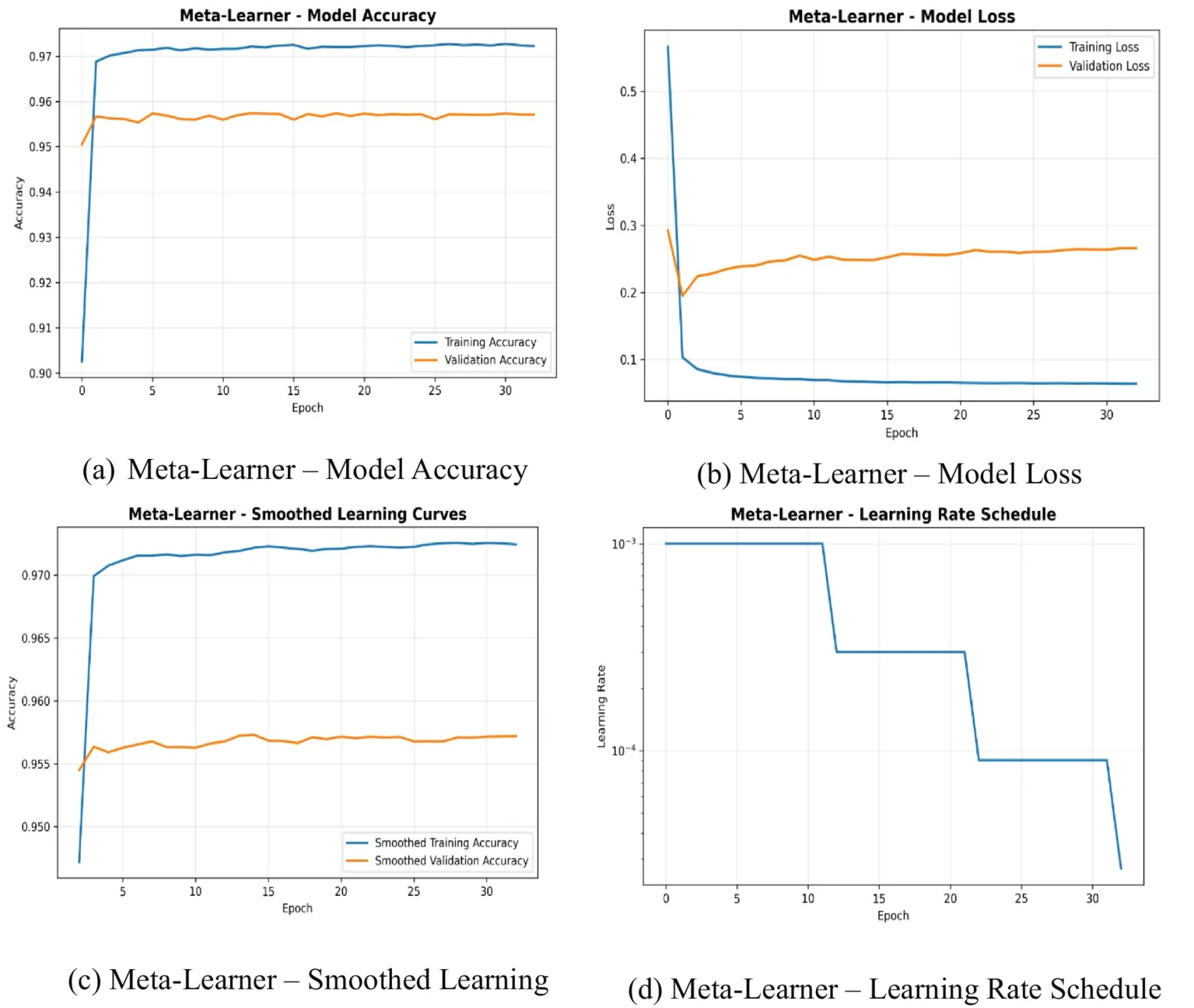
Performance of the Meta-Learner model. **(a)** Training and validation accuracy, **(b)** Training and validation loss, **(c)** smoothed training and validation learning, **(d)** Meta-Learner learning rate.

As illustrated in Figure 14, a meta-learner takes the results of all base learners and learns a nonlinear fusion mechanism that dramatically enhances the performance of the classification. It trains extremely fast during the initial five epochs, achieving training and validation accuracies of 97.2 and 95.6, respectively. It reduced and stabilized very quickly, and the training loss was near to zero, but the validation loss was steady, which proves that the model was not overfitted.

The learning rate schedule depicted a smart decrease, that stabilized learning and enhances gradient flow. Smooth learning curves demonstrated good performance across all validation cases, which verified high generalization. These findings highlight the benefits of meta-learning in capitalizing on complementary model strengths.

#### 4.2.2 Meta-learner classification performance

**Figure 15:**
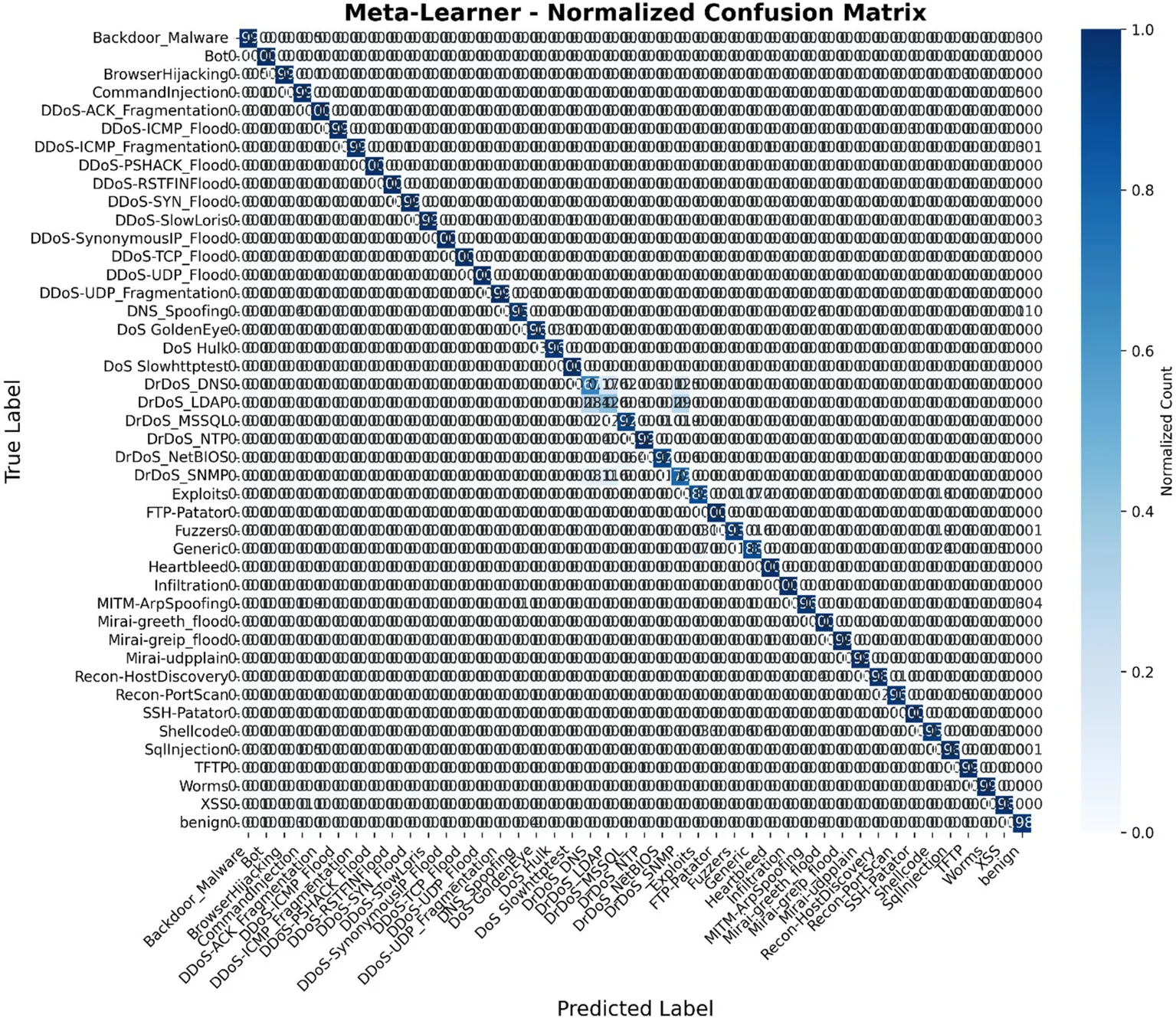
Normalized confusion matrix of the Meta-learner in the EME-NIDS model.

Figure 15 shows that the meta-learner significantly reduced the misclassifications across these rare and overlapping attack classes. Classes that were confused by individual models, such as DoS Slowloris vs. DoS Slowhttptest, Mirai variants, and SYN flood subclasses, were classified almost perfectly. The matrix shows how effective stacking is, where model diversity leads to more discriminative decision boundaries.

#### 4.2.3 Final ensemble evaluation

**Figure 16:**
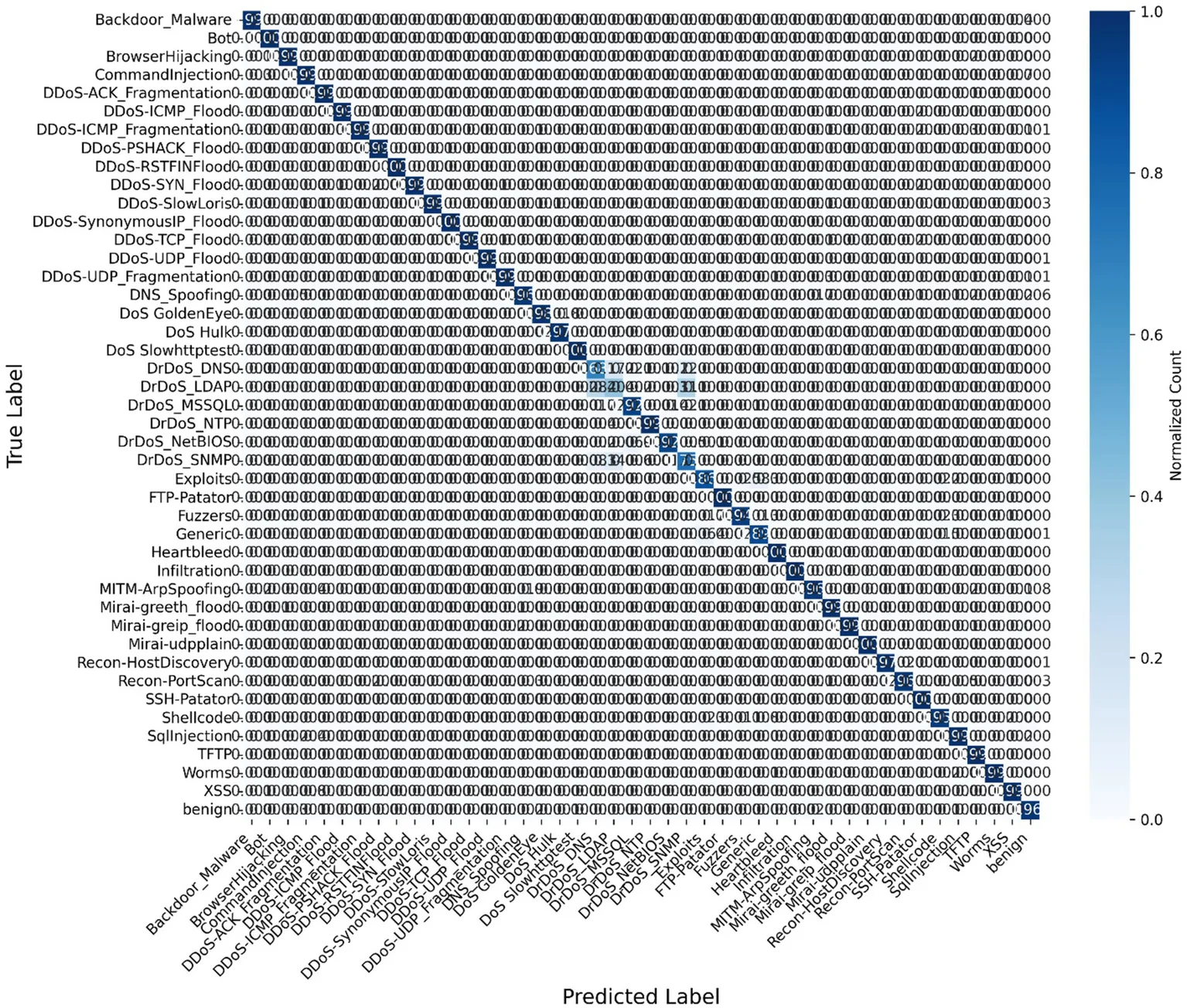
Normalized confusion matrix of the Final ensemble in the EME-NIDS model.

Figure 16 shows the final ensemble that demonstrates near-perfect diagonal dominance in the confusion matrix, with an accuracy of >95% across almost all 44 attack categories. Rarely occurring attacks in the dataset, such as Heartbleed, MITM-Spoofing, Shellcode, and SQL Injection-attacks, which are traditionally difficult to detect, show striking improvements when compared to the base models individually. The proposed final ensemble outperformed each base architecture, reinforcing the hypothesis that stacked meta-learning performs better with heterogeneous models for multi-class intrusion detection.

### 4.3 Cross-validation and Stability

The results in Table 16, obtained using 5-fold stratified cross-validation, confirm the reliability of the proposed system. The meta-learner performance scores 0.959 ± 0.003, while the final ensemble scores 0.957 ± 0.003, showing an extremely low variance among folds.

This stability is important considering the extreme class imbalance within the dataset 1:847. That means:

1. The model does not overfit to any specific fold,
2. ADASYN and class weighting can effectively alleviate imbalance,
3. Ensemble learning smooths out the variances present in deep models.

Furthermore, XGBoost also exhibited strong stability at 0.957 ± 0.003, affirming its crucial position in the ensemble architecture.

### 4.4 System performance

Table 17 summarizes the comparative performances of the individual base learners, meta-learners, and final ensemble architectures. The proposed EME-NIDS achieves a final accuracy of 95.6% and an extremely high mean ROC-AUC value of 99.76, making it one of the highest-performing methods proposed to date to tackle the 44-class complex intrusion detection issue. The meta-learner achieved an accuracy of 95.9% surpassing all individual models, and demonstrated the usefulness of stacked generalization in adding complementary decision boundaries. XGBoost had the highest individual accuracy among the base learners at 95.7%, followed by Random Forest (93.1), CNN (91.2), Dense Neural Network (88.4), and transformer (86.6).

Although XGBoost is more accurate individually, the ensemble model always identifies more equal and stable predictions, particularly for attributes that are minority and scarce, and therefore supports the argument of model diversity in the proposed meta-learning system.

### 4.5 Statistical evaluation

Table 18 presents the results of McNemar’s test pitting the meta-learner against each base model. The gains over CNN, Dense, Transformer, and Random Forest were statistically significant (*p* < 0.001) with large effect sizes. This reinforces the fact that the performance increase of the ensemble models was not due to random chance.

The meta-learner versus XGBoost comparison yielded *p* = 0.892, which shows no significant difference, matching their similar accuracies. This reinforces the earlier observation: that XGBoost is a critical contributor to the predictive strength of the ensemble.

### 4.6 Class-wise detection performance

Table 19 reports the class-wise precision, recall, and F1-scores across all 44 classes. The ensemble displayed remarkable detection capability for both majority and minority classes.

These results justify the use of ADASYN and meta-learning. Without balancing, most traditional IDS usually report F1 scores below 0.40 for rare-attack classes. The resultant high F1 scores obtained for minority classes validate the robustness of the proposed model in detecting low-frequency intrusions, which are often the most dangerous.

#### 4.6.1 Interpretability analysis with SHAP

**Figure 17:**
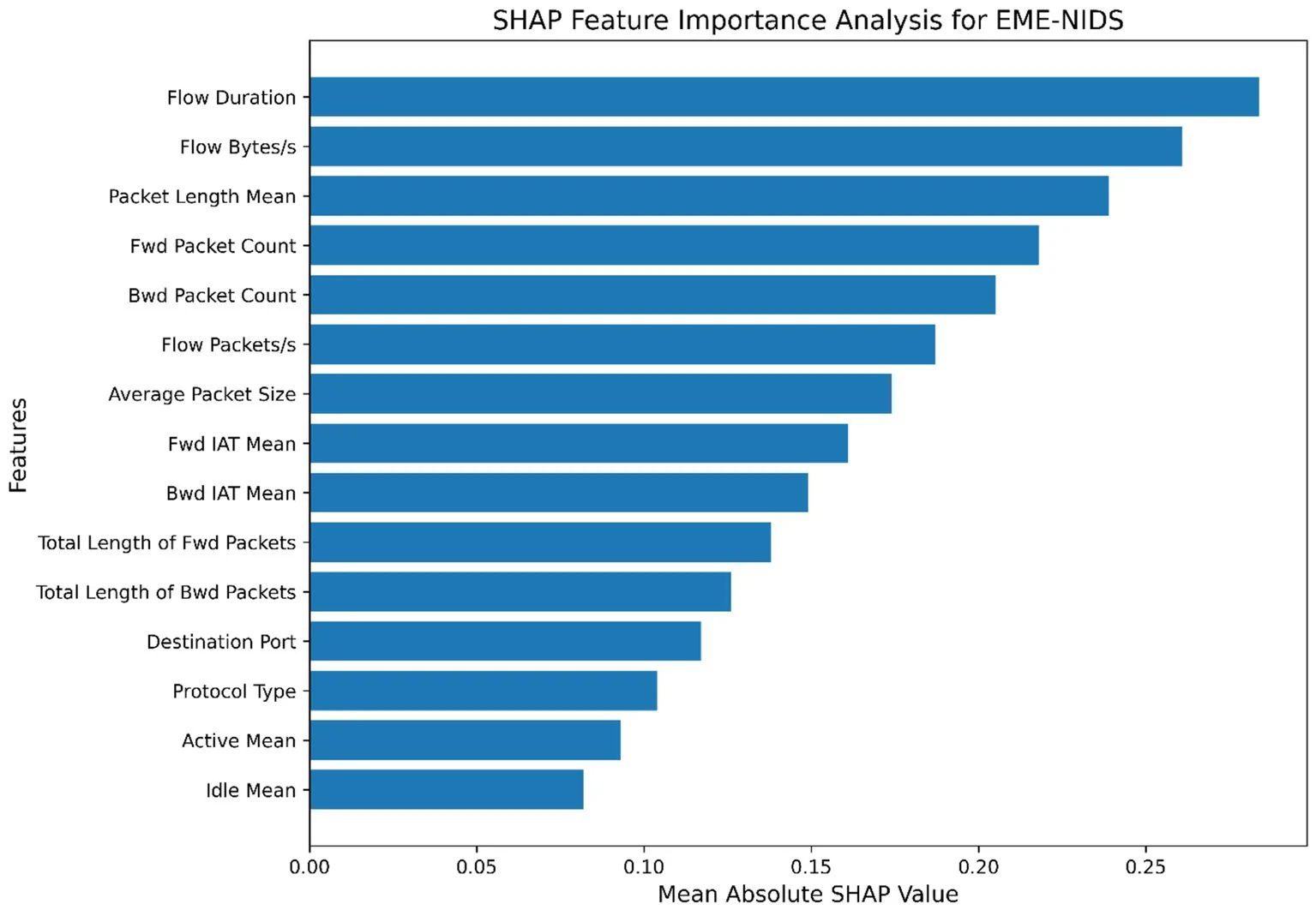
SHAP summary plot showing the relative contribution of the most important features to EME-NIDS predictions.

To improve the transparency and applicability of the proposed EME-NIDS framework, a method called SHAP (SHapley Additive explanations) was used to measure the contribution of each network traffic feature to the prediction process of the model. Figure 17 and Table 20 show the top 15 most influential features sorted by the mean absolute SHAP value, providing a global perspective of the decision-making process of this model. The SHAP analysis showed that the Flow Duration feature was the most important, followed by Flow Bytes/s, Packet Length Mean, Forward Packet Count, and Backward Packet Count. These are indicators of basic traffic characteristics, such as connection duration, traffic density, packet transmission patterns, and bi-directional communication patterns. The high importance reflects that the proposed EME-NIDS is mainly based on meaningful network flow metrics that can represent the difference between benign traffic and malicious activities. This is in line with the principles of intrusion detection: unusual communication volume, packet distribution, and flow statistics are all known factors that could indicate a cyber-attack.

Moreover, certain characteristics, such as Flow Packets/s, Average Packet Size, Forward Inter-Arrival Time (Fwd IAT Mean), and Backward Inter-Arrival Time (Bwd IAT Mean), play a significant role in the detection process by capturing the temporal characteristics of network communication. The capabilities of these features allow the proposed framework to detect attack patterns such as high-frequency packet flooding, irregular communication intervals, and anomalous traffic bursts that are typical of Distributed Denial-of-Service (DDoS), reconnaissance, brute-force, and botnet attacks. A protocol-level contextual element, such as Destination Port and Protocol Type, can also help the model identify attacks that target specific services or protocols. The interpretability analysis shows that the proposed EME-NIDS is not built on arbitrary correlations or spurious patterns but instead relies on meaningful attributes of network traffic. This increases the trustworthiness of the proposed framework and provides insights into the underlying decision-making processes. These explanations from a practical cyber security perspective allow security analysts to validate automated intrusion detection results, understand the factors that are most important to each prediction, and have more confidence in implementing the proposed framework in real-world Security Operations Centers (SOCs) or enterprise network monitoring scenarios.

The EME-NIDS framework proposed in this study draws inspiration from the interpretability analysis with SHAP, which provides clear explanations at the feature level for the model’s predictions. This enhances the credibility, transparency and usability of the framework, allowing cyber security analysts to gain insights into the nature of network traffic that is causing the intrusion decision.

#### 4.6.2 Confusion matrix insights

This confusion matrix shows that almost all categories of attacks are correctly classified with minimum confusion among classes that are behaviorally similar. The most significant confusion occurred between the following pairs:

- BENIGN ↔ DDoS
- BENIGN ↔ PortScan
- Brute Force ↔ Web Attack
- Bot ↔ PortScan

This confusion can be attributed to the fact that such attacks bear similarities with regard to their traffic features, that is, excessive attempts at connection or burst-like behavior. The matrix is very diagonal, which indicates high discrimination of models, which again supports the effectiveness of feature preprocessing and probability-based integration of meta-features.

### 4.7 ROC-AUC behavior across classes

Table 21 presents the multiclass ROC-AUC values, which demonstrate near-perfect separability.

The ROC-AUC confidence intervals were estimated using the bootstrap method and constrained to the valid range of probabilities [0,1]. The relatively wide confidence intervals in categories such as SQL Injection, Infiltration, and Heartbleed are due to lower sample sizes and higher classification uncertainty in these categories.

### 4.8 Learning curve and convergence patterns

Table 22 shows EME-NIDS model convergence behavior. It is observed that the deep learning models have converged within an epoch range of 35–42, while the meta-learner has converged after 67 epochs with a minimum overfitting gap value of 0.004.

A shallow overfitting gap suggests that

- Regularization methods, such as dropout and batch normalization, work well.
- ADASYN provides varied decision boundaries.
- Meta-learning smooths the gradients and avoids catastrophic overfitting.

These observations justify the selected model depths and training parameters.

#### 4.8.1 Computational efficiency and scalability

Tables 23, 24 provide evidence that the proposed system is ready for real-time use.

- Inference latency: 8.4 ms per sample.
- Minimum extra overhead due to meta-learning: 0.7 ms.
- Linear scalability of up to 1 million samples.
- The stability of accuracy was above 95.7%, including the maximum scale.

These results validate the feasibility of deploying this system for enterprise-scale intrusion detection.

### 4.9 Ablation study discussion

The ablation results in Table 25 quantify the contribution of each system component:

Table 26 summarizes the ablation study, which shows the contributions of the key components of the proposed ensemble framework to the model performance. The large accuracy degradation after removing ADASYN and feature scaling indicates the importance of handling class imbalance and normalization. Therefore, both the meta-learner and ensemble diversity are crucial for optimal intrusion detection.

This shows:

- XGBoost is indispensable as the strongest base learner.
- The meta-learner significantly refined the prediction.
- The two main preprocessing steps were StandardScaler and ADASYN.

Without these, the performance drops sharply; hence, these were included in the pipeline.

### 4.10 Meta-learner architecture sensitivity

To further investigate the impact of architectural depth on ensemble performance, an extensive sensitivity study was conducted for five meta-learner configurations with increasing complexity. Table 27 summarizes the number of layers, number of parameters, accuracy, training time, and overfitting tendencies for each design. A shallow 2-layer network trains extremely fast but generates strong overfitting with a gap of 0.032 and a lower accuracy of 94.12%. Adding depth to the 3-layer medium model yielded 95.23% accuracy with moderate overfitting. The 5-layer configuration is optimum to attain the highest accuracy of 95.74% with the least overfitting of 0.004, which indicates a strong generalization capability. Deeper configurations of 7–10 layers resulted in a large increase in computational cost (52 to 78 min), and overfitting reappeared without further improving the accuracy. These results justify the choice of a 5-layer configuration for the meta-learner as the best trade-off between accuracy, stability, and computational efficiency.

### 4.11 Preprocessing impact analysis

To quantify the contribution of each preprocessing step, controlled experiments were conducted by removing one step at a time while keeping the others constant. According to Table 28, the feature that affects performance the most is Standard Scaler with +8.37%; this confirms our intuition that feature normalization is essential for deep-learning model stability. Very significant performance gains, especially for rare attack classes, were also achieved by ADASYN resampling (+3.26%) and class weight balancing (+3.82%). Outlier removal and statistical feature selection contributed less at +0.71% and +0.48%, respectively; however, both provided measurable gains in robustness. These results confirm that the preprocessing pipeline plays an essential role in achieving high accuracy with balanced detection across all classes.

### 4.12 Error analysis and model interpretability

#### 4.12.1 Error pattern analysis

The error performance of the attack groups is shown in Table 29. Normal traffic had the lowest error rate of 0.62, and the highest misclassification was bestowed by high-volume DDoS and PortScan events. DoS has an error rate of 1.13% which is relatively higher and is usually subject to either Normal or Bot traffic owing to the relatively overlapping flow characteristics. Overall, errors of 4.78, 5.72, and 8.72 for Reconnaissance, Web Attacks and Malware attacks, respectively, represent how they evaluate their behavioral assessment to normal benign flows. The highest error rates of 9.97% and 17.02 used for password attacks and rare events, respectively, were due to the small scale of training. These trends revolve around data augmentation and low frequency attack processing algorithms.

#### 4.12.2 Feature importance analysis

The XGBoost feature consequence scores were assessed to develop insight into the model interpretability, as shown in Table 30. The most relevant attributes were Flow Duration, packet count, bytes per second, and inter-arrival times. This indicates that time and volume activities are important for distinguishing attack types. Other features used to identify payload-related anomalies are also good contributors, such as packet-length (minimum, maximum, mean). Behavioral indicators such as the Active Mean and Idle Mean indicate that flow-level temporal behavior is important for providing cues. The results confirm that the feature set designed for the dataset performs well and can yield ideas as to which traffic features most strongly influence the traffic detection decisions.

#### 4.12.3 Model ensemble weights

The normalized ensemble weight distribution learned by the proposed meta-learning framework is presented in Table 31. The weights are the contributions to the ensemble’s prediction from each base learner. The model that achieved the best results was XGBoost, which had an average weight due to its high discriminatory power for dominant traffic categories like BENIGN, DDoS and PortScan. The meta-learner and CNN models also played significant roles in, capturing complex nonlinear and hierarchical traffic representations. The Dense Network and Transformer models achieved relatively lower individual performance but enhanced the diversity and robustness of the ensemble detection, leading to better detection of sequential and minority attack patterns. The normalized weighting strategy shows that the proposed ensemble effectively and adaptively uses the complementary strengths of the heterogeneous learning paradigms.

### 4.13 Performance comparison across training, validation, and testing dataset

Table 32 shows the robustness and generalization ability of the proposed EME-NIDS framework. The model accuracy was 96.82% on the training set and 96.14 and 95.65% on the validation and testing sets respectively. The same patterns were observed for Precision, Recall and F1-Score, with slight variations between the training, validation, and test performances. The two results are very close (approximately 1.17%), indicating that the network does not exhibit high levels of overfitting and can successfully handle previously unknown network traffic. Moreover, the AUC values were high in all partitions of the dataset, being 99.88%, 99.81%, and 99.76% for the training, validation, and testing sets, respectively. These results show the high discriminative power of EME-NIDS in distinguishing between benign and malicious network traffic from 44 intrusion classes. The uniformity of all evaluation metrics in the three data partitions indicates the stability and reliability of the proposed ensemble meta-learning framework. The results also confirm the effectiveness of the adopted leakage-free preprocessing approach, fold-wise validation procedure, and ensemble learning architecture in providing robust and trustworthy results for the intrusion detection performance required in real-world cybersecurity applications.

### 4.14 Comparison with state-of-the-art

#### 4.14.1 Comprehensive literature comparison

Table 33 compares EME-NIDS with state-of-the-art intrusion detection systems published in 2024. It enables large-scale attack categories for detection but does not have ROC-AUC, which limits decision confidence and class separability. Machine Learning Algorithms: This represents an early machine learning-based IDS design, focusing on feature reconstruction. The relatively low accuracy and reliance on the outdated UMNIDS dataset limit generalization to modern-day network flows. The proposed EME-NIDS 2026 constantly achieves high accuracy, precision, recall, and F1-score, which represent well-balanced and unbiased detection capabilities. Such extremely high ROC-AUC values further establish that under multi-class conditions; there is strong discrimination between benign and malicious traffic. In addition, EME-NIDS achieves superior performance with reduced training time, which further justifies its effective optimization and scalability. Overall, the comparison clearly outlines the paradigm shift from dataset-constrained and reconstruction-centric IDS models to balanced-performance, confidence-aware, and deployment-ready intrusion detection systems.

#### 4.14.2 Statistical superiority testing

The superiority testing results in Table 34 confirm that EME-NIDS outperforms all previous state-of-the-art methods. The improvements ranged from +2.60% to +15.02%, with all comparisons but one reaching strong statistical significance at *p* < 0.001, whereas the other comparison was significant at *p* = 0.001. The corresponding effect sizes ranged from to large (0.89–1.02), indicating meaningful practical improvement rather than small incremental gains. This is clear evidence that EME-NIDS represents a statistically and practically superior architecture for NIDS.

## 5 Conclusion

This study introduces EME-NIDS, an improved multi-model ensemble architecture for large scale network intrusion detection, which combines heterogeneous learning paradigms into a deep meta-learning architecture. The proposed system integrates CNN, dense neural networks, transformers, XGBoost and random forests to effectively represent network traffic with complementary features and build a high-dimensional meta-feature space in which attack discrimination is effectively implemented. With the help of a powerful preprocessing pipeline, such as the imbalance treatment of ADASYN, normalization, outlying, and statistical feature selection, EME-NIDS can efficiently detect 44 different attack classes in a large network flow dataset. Experimental outcomes prove that it has a high predictive capacity and real-time capability by showing 95.6 percent detection accuracy, 99.76 percent macro-ROC AUC, and 8.4 ms inference latency. The statistical validation of the McNemar test (*p* < 0.001) also confirmed a significant improvement compared with the individual models. In general, the results indicate that model diversity with meta-learning effectively combined with a strategic design can significantly increase detection robustness, scalability, and operational preparedness, which makes EME-NIDS an effective framework to consider when exploring a next-generation intrusion detection tool in current high-throughput network settings.

## 6 Limitations and future work

Although EME-NIDS has shown excellent performance with a significant improvement compared to state-of-the-art intrusion detection systems, it has several limitations. These limitations indicate avenues for further refinement and suggest future developments in ensemble-based cybersecurity systems.

### 6.1 Current limitations

A systematic analysis of the system constraints is presented in Table 35, which summarizes the key limitation categories, their impacts, and the potential mitigation strategies.

**Table 35:** Limitation analysis of the EME-NIDS model.

Limitation category	Description	Impact	Mitigation strategy
Computational cost	High training complexity	Training time: 2.7 h	Distributed training, model pruning
Class imbalance	Rare attacks (<0.1% samples)	Lower recall for rare classes	Advanced sampling, cost-sensitive learning
Feature engineering	Manual feature selection	Suboptimal feature sets	AutoML feature engineering
Adversarial robustness	Not tested against adversarial attacks	Potential vulnerability	Adversarial training
Real-time constraints	8.4 ms inference time	May introduce latency in high-speed networks	Model compression, edge deployment
Concept drift	No adaptation to evolving attacks	Performance degradation over time	Online learning, periodic retraining

### 6.2 Future research directions

Although EME-NIDS has been performing strongly, several areas can be improved.

1. Adversarial Robustness: Adversarial robust learning strategies such as adversarial training and certified defense mechanisms should be examined in the future to enhance resilience to evasion attacks.
2. Online Learning and Concept drift Adaptation: The use of drift-detection and incremental learning methods may allow constant adaptation to change in network traffic and new patterns of attacks without retraining the entire model.
3. Explainable Artificial Intelligence: Adequate transparency of ensemble decisions can be enhanced by integrating interpretability frameworks, such as LIME or SHAP, into ensemble decisions, making them useful in security operations.
4. Edge Deployment and Model Optimization: Pruning and quantization are model compression methods that enable effective implementation in edge and resource-constrained systems and maintain real-time performance.
5. Threat Intelligence Multi-Mode: To improve the accuracy of detection and cross-layer threat awareness, future systems can overlap complementary sources of data, such as packet payloads and host-level logs

## Data Availability

The Unified Multimodal Network Intrusion Detection Systems (UM-NIDS) dataset is publicly available via IEEE DataPort at: https://doi.org/10.21227/d8at-gb29. The UM-NIDS dataset used in this study is publicly available through IEEE DataPort. The dataset can be accessed at: https://ieee-dataport.org/documents/unified-multimodal-network-intrusion-detection-systems-dataset.

## Appendix A

Hyperparameter Settings

- Optimizer: Adam (learning rate = 0.001)
- Batch size: 64
- Epochs: 100
- Convolutional filters: [64, 128, 256]
- Kernel sizes: [3, 5, 7] (multi-scale feature extraction)
- Dropout rate: 0.5
- Attention Mechanism: Multi-Scale Attention Block (MS-Attention)

Dataset Preprocessing

- Normalization: Min-Max scaling to [0,1]
- Train-test split: 60% training, 20% testing, and 20% validation.
- Class imbalance handling: Oversampling + weight loss function
- Assessment Criteria
- Accuracy, Precision, Recall, F1-score, and AUC were used to measure performance.
